# Chimeric TIM-4 receptor-modified T cells targeting phosphatidylserine mediates both cytotoxic anti-tumor responses and phagocytic uptake of tumor-associated antigen for T cell cross-presentation

**DOI:** 10.1016/j.ymthe.2023.05.009

**Published:** 2023-05-16

**Authors:** Brandon Cieniewicz, Ankit Bhatta, Damoun Torabi, Priya Baichoo, Mike Saxton, Alexander Arballo, Linh Nguyen, Sunil Thomas, Harini Kethar, Phanidhar Kukutla, Omolola Shoaga, Bi Yu, Zhuo Yang, Maria Fate, Edson Oliveira, Hongxiu Ning, Lawrence Corey, Daniel Corey

**Affiliations:** 1 Cero Therapeutics Inc, South San Francisco, CA 94080, USA; 2 Vaccine and Infectious Disease Division, Fred Hutchinson Cancer Research Center, Seattle, WA 98109, USA

**Keywords:** adoptive T cell therapy, phagocytic clearance, epitope spread, oncology/hematology, combination therapies, EGFR mutation-positive NSCLC, BTK inhibitor therapy, chimeric engulfment receptor, engineered T cells, chimeric antigen receptor

## Abstract

To leverage complementary mechanisms for cancer cell removal, we developed a novel cell engineering and therapeutic strategy co-opting phagocytic clearance and antigen presentation activity into T cells. We engineered a chimeric engulfment receptor (CER)-1236, which combines the extracellular domain of TIM-4, a phagocytic receptor recognizing the “eat me” signal phosphatidylserine, with intracellular signaling domains (TLR2/TIR, CD28, and CD3ζ) to enhance both TIM-4-mediated phagocytosis and T cell cytotoxic function. CER-1236 T cells demonstrate target-dependent phagocytic function and induce transcriptional signatures of key regulators responsible for phagocytic recognition and uptake, along with cytotoxic mediators. Pre-clinical models of mantle cell lymphoma (MCL) and EGFR mutation-positive non-small cell lung cancer (NSCLC) demonstrate collaborative innate-adaptive anti-tumor immune responses both *in vitro* and *in vivo*. Treatment with BTK (MCL) and EGFR (NSCLC) inhibitors increased target ligand, conditionally driving CER-1236 function to augment anti-tumor responses. We also show that activated CER-1236 T cells exhibit superior cross-presentation ability compared with conventional T cells, triggering E7-specific TCR T responses in an HLA class I- and TLR-2-dependent manner, thereby overcoming the limited antigen presentation capacity of conventional T cells. Therefore, CER-1236 T cells have the potential to achieve tumor control by eliciting both direct cytotoxic effects and indirect-mediated cross-priming.

## Graphical abstract

**Figure:**
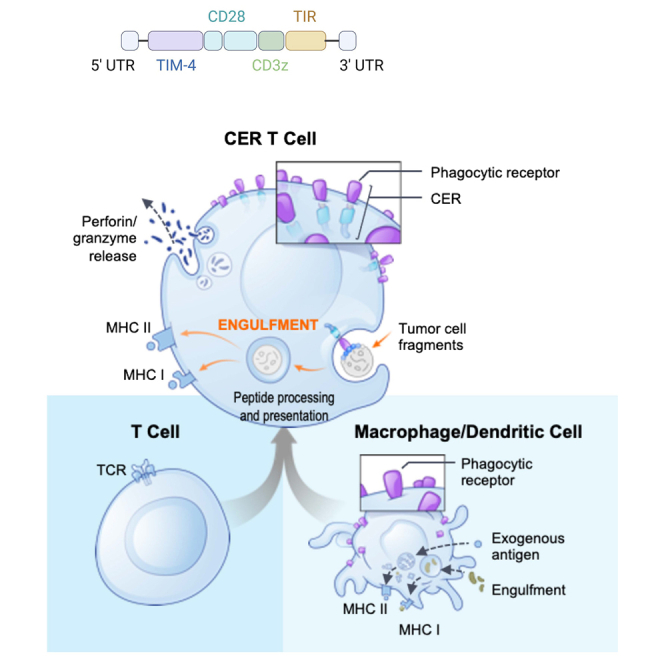

## Teaser

Corey and colleagues present a novel autologous cell therapy in which T cells are engineered with a chimeric engulfment receptor that targets cancer cells’ “eat me” signal. This combines direct cell killing and phagocytic antigen presentation into single T cells to achieve tumor control in solid and hematologic tumors.

## Introduction

Engineered T cells have emerged as a powerful cancer therapy, yet multiple barriers exist that limit their efficacy.^1^^,^^2^^,^^3^^,^^4^ A complementary cell clearance approach involves harnessing innate immune effector responses, including phagocytic clearance of cancer cells and induction of vaccine-like anti-tumor responses.^5^^,^^6^^,^^7^ Here, we evaluate an engineering and therapeutic strategy designed to co-opt phagocytic clearance and antigen presentation activity into T cells to create chimeric engulfment receptor (CER)-T cells for cancer immunotherapy.

The recognition of phagocytosis as a therapeutic modality to directly clear cancer cells and initiate anti-tumor T cell immune responses has fueled interest to effectively engage phagocytes for tools and targets in cancer therapy. Macrophage cell engineering (CAR-M) and macrophage-targeting approaches that enhance cytotoxic, phagocytic, and cytokine-mediated anti-tumor function are in development, and early clinical trial data from therapeutics targeting myeloid inhibitor function shows potential to elicit clinical responses.^8^^,^^9^^,^^10^^,^^11^^,^^12^^,^^13^^,^^14^^,^^15^ However, the diverse pro-tumor functions of myelo-monocytic cells may counterbalance these efforts by supporting cancer cell survival, proliferation, and release of factors that may impede anti-tumor immune responses.^16^^,^^17^^,^^18^^,^^19^^,^^20^^,^^21^ Limited *in vivo* proliferation and manufacturing challenges have also been hurdles in the development of mononuclear phagocyte-based cellular therapy.^22^

Although phagocytosis has traditionally been associated with myeloid cells, it has also been observed in various other cell types in both normal and pathological contexts.^23^^,^^24^^,^^25^^,^^26^^,^^27^^,^^28^^,^^29^ To take advantage of this phenomenon, we engineered CER-1236, a chimeric receptor that fuses the external domain of TIM-4, a phagocytic receptor, with intracellular signaling domains from T cells and innate immune cells. TIM-4 harbors endogenous phagocytic capacity via binding to the pro-phagocytic “eat me” signal, phosphatidylserine (PS), and has been linked to uptake of tumor-associated antigens and anti-tumor responses.^30^^,^^31^^,^^32^^,^^33^ Ectopic expression of TIM-4 on heterologous cells has also been shown to render these cells capable of internalizing PS-bearing target cells by utilizing integrin coreceptors to activate phagocytic signaling.^30^^,^^34^ CER-1236’s intracellular signaling domains, including Toll-like receptor 2 (TLR2)/Toll/interleukin (TIR), CD28, and CD3ζ motifs, are designed to augment both TIM-4-mediated phagocytosis and T cell cytotoxic function. TLR2/TIR has been shown to activate RAC1 GTPase, which acts as a key signal transducer in phagocytosis.^35^

We tested the combined cytotoxic and phagocytic function of CER-1236 T cells in pre-clinical models of lymphoma and EGFR mutation-positive non-small cell lung (NSCLC) adenocarcinoma. In normal tissues, PS is confined to the internal leaflet of the plasma membrane. By contrast, hematologic and solid tumor cell lines and primary and metastatic tumors display cell surface PS, which can be further augmented using standard-of-care therapies via induction of cell damage and death.^36^^,^^37^ Enhancement of tumor immunogenicity was also evaluated in antigen cross-presentation assays. Compared with conventional T cells, activated CER-1236 T cells have the ability to efficiently process and display antigens, thus overcoming T cells’ limited antigen presentation capacity caused by inefficient antigen capture.^38^^,^^39^

## Results

### CER-1236 is designed to elicit multiple functions via its TIM-4 phagocytic receptor together with innate and adaptive intracellular signaling domains

**Figure 1:**
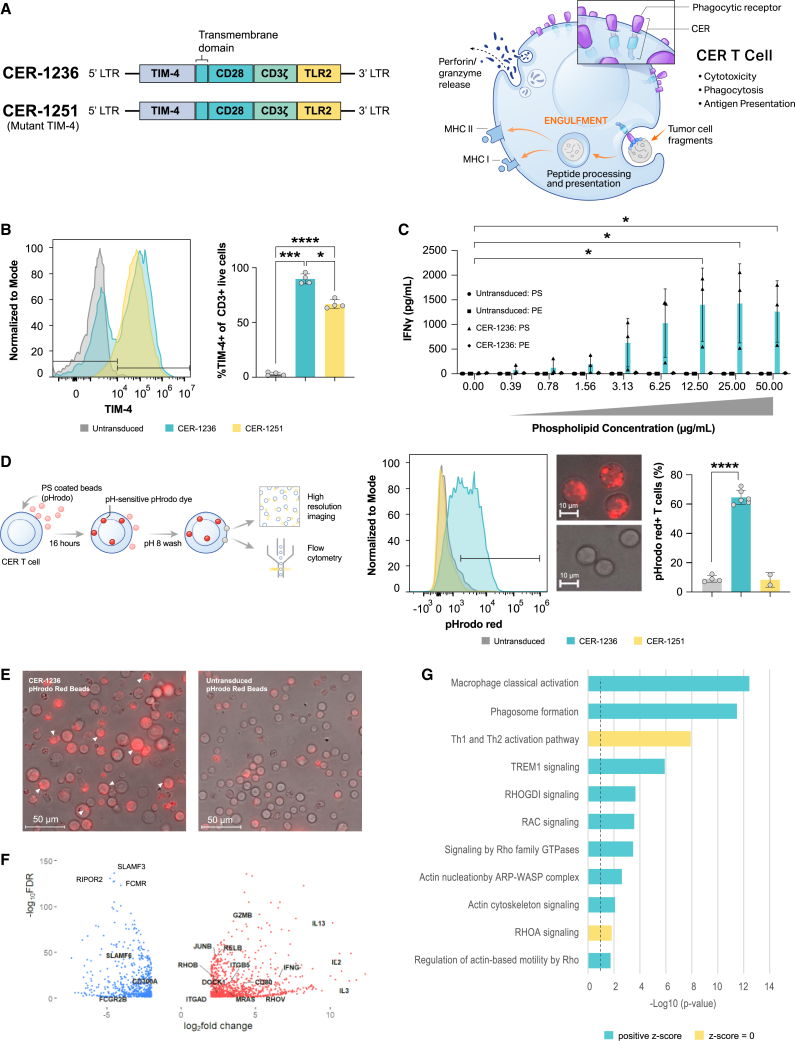
Generation of CER-T cells, TIM-4-expressing T cells, activated in response to PS, can phagocytose targets, and upregulate phagocytic gene modules (A) Schematic of CER-1236 expression construct and the CER-1251 PS binding site mutant variant. Expression of CER-1236 allows for target-dependent T cell activation and phagocytosis of antigen-positive target. (B) Transduction of transduced T cells, as measured by expression of the TIM-4 extracellular domain, was determined at the end of production by flow cytometry. A representative histogram is shown, and average transduction ± SEM is shown (n = 4 donors). Significance was determined using a one-way ANOVA with Geisser-Greenhouse correction with Tukey’s multiple comparisons test. ∗p < 0.05, ∗∗∗p < 0.001, ∗∗∗∗p < 0.0001. (C) IFN-γ secretion in response to immobilized PS. IFN-γ secretion was measured in the supernatant of cells 24 h after plating on the indicated amount of PS or PE by automated ELISA. Average ± SEM is shown (n = 3 donors). ∗p < 0.05. (D and E) CER-1236 phagocytosed PS-coated beads. CER-1236 T cells were incubated with PS-coated agarose beads labeled with the pH-sensitive dye pHrodo Red. After 16 h, phagocytosis was measured by flow cytometry, as determined by pHrodo Red^+^ T cells. The average ± SEM is shown (n = 4 for untransduced, 6 for CER-1236, and 2 for CER-1251). Parallel samples were imaged by fluorescence microscopy at 40×. Representative images of CER-1236 or untransduced cells at 40× are shown in (D). Representative images at 4× are shown in (E). (F) CER-1236 upregulate both T cell activation and pro-phagocytic genes. RNA sequencing was performed on CER-1236 T cells, TIM-4 mutant 1251 T cells, or untransduced control T cells stimulated with plate bound PS for 24 h. A volcano plot of genes with an absolute fold change > 4, FDR < 0.05 are shown. (G) IPA pathway gene sets (FDR < 0.05, absolute fold change > 4) comparing 1236 with TIM-4 mutant 1251 from three donors 24 h post-stimulation. CER, chimeric engulfment receptor; TIM-4, T cell immunoglobulin and mucin domain containing 4; TLR, Toll-like receptor; LTR, long terminal repeat; MHC, main histocompatibility complex; IFN, interferon; PS, phosphatidylserine; PE, phosphatidylethanolamine.

We fused the pro-phagocytic receptor extracellular domain TIM-4 with CD28, CD3ζ, and TLR2 TIR-1 receptor intracellular signaling domains (Figure 1A, left). Previous studies established that expression of TIM-4 on non-myeloid cells renders them capable of internalizing targets, and that its transmembrane and cytoplasmic domains are dispensable for its function in phagocytosis.^30^^,^^31^^,^^34^ TIM-4 recognizes PS and is predominantly expressed on resident peritoneal macrophages and some subsets of dendritic cells, where it has been shown to mediate uptake of tumor-associated antigens and initiate anti-tumor responses.^30^^,^^32^^,^^33^^,^^40^ CER-1236 exploits the phagocytic and PS-binding properties of TIM-4 and T cell activation capacity of CD3ζ and CD28 (Figure 1A, right). The activation of TIR further enhances signaling through both NF-κB and the mitogen-activated protein kinase family, promoting T cell activity, and phagocytic uptake by activating actin-Cdc42/Rac pathways.^35^^,^^41^^,^^42^ CER-1236 T cells were produced by transducing donor T cells with a lentiviral vector encoding CER-1236, resulting in a high proportion of transduced TIM-4 receptors, high viability over the course of cell production, robust expansion, similar CD4:CD8 ratios to that of untransduced T cells, similar levels of PD-1 expression over the course of production, and high levels of CCR7^+^ naive and central memory T cells (Figures 1B and S1A–S1E). A negative control, CER-1251 T cells, with matched intracellular signaling domains but unable to bind to the PS target due to TIM-4 binding site mutation, were also prepared.

We developed a cell-free *in vitro* activation system using a titrating density of plate-bound target antigen to functionally characterize antigen-driven CER-1236 cytokine responses. CER-1236 T cells secreted IFN-γ in a dose-dependent manner in response to plate-bound PS (Figure 1C), peaking at 12.5 μg/mL. CER-1236 T cells showed minimal IFN-γ secretion in response to high-dose plate-bound phosphatidylethanolamine (PE), a related phospholipid used as a control, or when plated on uncoated wells, demonstrating the specificity of CER-1236 T cells to PS. Untransduced and CER-1251 T cells secreted <5 pg/mL in all conditions. Blockade of signaling through the TLR2 domain of CER-1236 using a small-molecule inhibitor C29 reduced secretion of granzyme B, IFN-γ, TNF-α, IL-2, IL-10, and IL-6 by CER-1236 cells but did not abrogate it (Figures S2A–S2F).^43^ Overall, these results demonstrate target-specific and dose-dependent IFN-γ production by CER-1236 in response to plate-bound target.

### CER-1236 demonstrates target-dependent phagocytic uptake and upregulates key phagocytic regulators

To quantify CER-1236 phagocytic potency, we developed an assay that utilizes PS-coated agarose beads pre-labeled with pHrodo Red, a pH-sensitive dye that minimally fluoresces at neutral pH but brightly fluoresces in acidic pH. The post-phagocytic fusion of phagosomes and lysosomes leads to a drop in pH, which can be detected by pH-sensitive dyes.^44^^,^^45^^,^^46^ Using this system, CER-1236 T cells co-cultured with PS-coated beads displayed robust phagocytic activity (Figure 1D), with over 60% of CER-1236 T cells acquiring a pHrodo Red signal, as measured by fluorescence-activated cell sorting (FACS). By contrast, untransduced T cells and mutant TIM-4 CER-1251 T cells showed minimal pHrodo Red signal, supporting a critical role for CER-1236 in bead internalization (Figure 1D). These findings were confirmed using fluorescence microscopy to assess the intracellular localization of PS beads for CER-1236 and untransduced T cells. Representative images from this analysis are presented (Figures 1E and S3), with multiple internalized beads evident per cell.

We performed bulk RNA sequencing to further interrogate the transcriptional profile of CER-1236 T cells after plate-bound stimulation. Principal-component analysis from three healthy donors observed a clear separation between 24 h CER-1236-activated groups and untransduced and TIM-4 mutant CER-1251 T cells (Figure S4). A total of 1,706 genes were differentially expressed (FDR < 0.05, absolute fold change > 4) in CER-1236-stimulated groups compared with TIM-4 mutant CER-1251-stimulated T cells (Figure 1F; Table S1). IPA pathway analysis identified 242 pathways –log(p) > 1.3 (Figure 1G; Table S2). Among these, were pathways well known to be involved in regulating phagocytosis, such as actin cytoskeleton signaling (DOCK1, RAC3, ITGB5, ITGAD), genes involved in nucleation of the ARP-WASP complex (RHOU, RHOV, RASD1), Rho family GTPases (CDC42EP1, GNAI1, RHOB, RELB), RAC signaling (CD44, CDK5R1, MRAS, NCF2), and phagosome formation (ADGRD1, ADGRD2, ADGRA3, TIMD4, C3, CR1, FCGR2B, CARD9, AP1S3) (Figures 1G and S4). The RhoG subfamily of GTPase, including MRAS has previously been implicated in T cell receptor (TCR)-driven phagocytic processes from antigen-presenting cell synapses.^47^

Negative regulators of phagocytosis were also some of the highest downregulated genes including CD300a, an inhibitory efferocytosis receptor, SLAMF5 and SLAMF3, inhibitory Ig family receptors, FCGR2B, an ITIM-containing inhibitory Fc receptor, FCMR, an inhibitory IgM Fc receptor, and signaling molecules Fgr and RIPOR2, negative regulators of phagocytosis through Src kinase and RhoA signaling, respectively. In line with CD28CD3ζ -containing motifs, T cell activation genes (IFN-γ, IL-3, CCL4, IL2RA), key cytotoxicity genes including GZMB (encoding granzyme B), and inflammatory cytokines (TNF-α, IL-2, IL-13, and GM-CSF) were also upregulated (Figures 1F and S4). Thus, taken together we identify a distinct transcriptional T cell signature in CER-1236 T cells that identifies well-known key regulators responsible for phagocytic recognition and uptake, along with cytotoxic mediators.

### *In vitro* cytotoxicity, cytokine production, and phagocytosis of CER-1236 T cells against MCL cell lines

**Figure 2:**
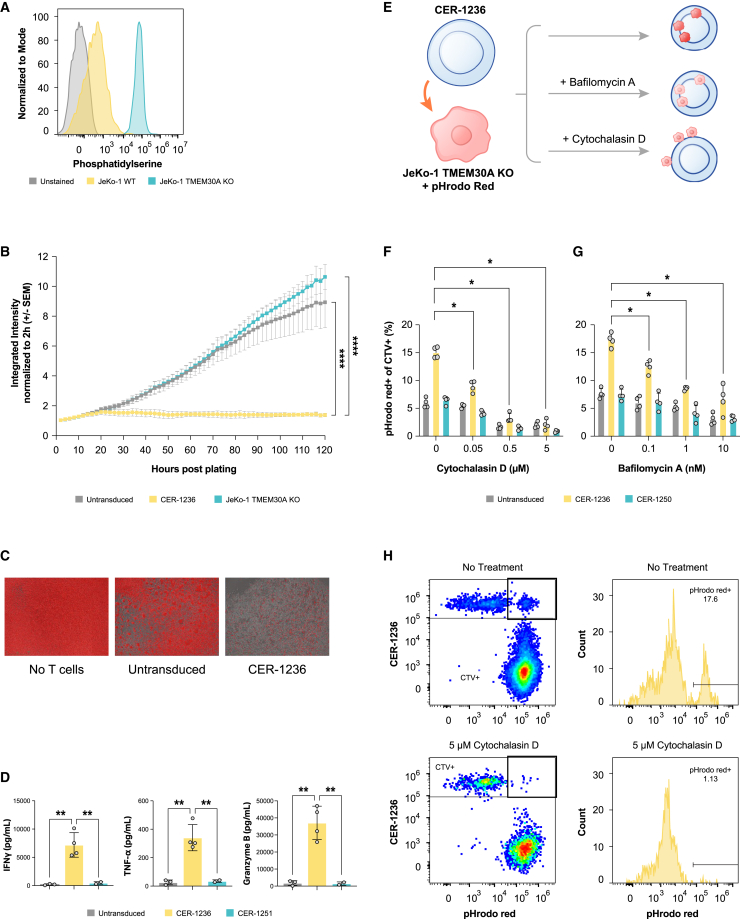
CER-1236 mediates cytotoxicity, cytokine secretion, and phagocytosis against PS-positive mantle cell leukemia cells (A) JeKo-1 mCherry^+^ TMEM30A KO cells have high constitutive expression of PS. PS exposure on JeKo-1 mCherry^+^ TMEM30A KO or JeKo-1 mCherry^+^ parental cells was measured by flow cytometry. Cells were stained with recombinant human TIM-4-His, then stained with secondary anti-HIS antibody. A representative histogram of PS staining is shown. (B) CER-1236 T cells potently kill PS^+^ target cells. CER-1236 T cells were co-cultured with JeKo-1 mCherry^+^ TMEM30A KO cells at a 1:1 ratio in medium supplemented with 200 IU/mL IL-2. Untransduced cells were included as controls. Cytotoxicity was measured by reduction in mCherry integrated intensity using the IncuCyte Live-Cell Analysis System. Average cytotoxicity ± SEM is shown (n = 4 donors). Statistics were analyzed at each time point using a two-way ANOVA with Tukey’s multiple comparisons test. Results for 120 h are shown. ∗∗∗∗p < 0.0001. (C) Representative IncuCyte image overlays of phase/contrast and red channel taken at 120 h post co-culture. mCherry^+^ cells are false colored red. (D) CER-1236 secrete effector cytokines in response to PS^+^ target cells. CER-1236 were co-cultured with JeKo-1 mCherry^+^ TMEM30A KO cells at a 1:1 ratio for 120 h. Untransduced cells were included as controls. After co-culture, cytokine secretion was measured by automated ELISA. Average cytokine secretion ± SEM is shown (n = 3 donors for untransduced, 4 donors for CER-1236, 2 donors for CER-1251). Statistics were analyzed using a one-way ANOVA with Tukey’s multiple comparisons test. ∗∗p < 0.01. (E) Schematic of phagocytosis assay setup. CER-1236 T cells were stained with CTV, and JeKo-1 mCherry^+^ TMEM30A KO cells were stained with pHrodo Red. T cells were co-cultured with target cells at a 1:2 ratio in the presence or absence of the phagocytosis inhibitors cytochalasin D or bafilomycin A, and incubated for 40 h in cytokine-free medium. At the end of co-culture, phagocytosis was measured by flow cytometry. (F) CER-1236 phagocytosis of JeKo-1 mCherry^+^ TMEM30A KO cells is impaired by cytochalasin D. Phagocytosis assays were performed with 5, 0.5, or 0.05 μM of cytochalasin D, an actin polymerization inhibitor. The average phagocytosis ± SEM is shown (n = 1 donor). (G) CER-1236 phagocytosis of JeKo-1 mCherry^+^ TMEM30A KO cells is impaired by bafilomycin A. Phagocytosis assays were performed with 10, 1, or 0.1 nM of bafilomycin A, an inhibitor of lysosome-phagosome fusion. The average phagocytosis ± SEM is shown (n = 1 donor). (H) Representative flow plot of phagocytosis in the presence or absence of cytochalasin D. SEM, standard error of the mean; TMEM30A, transmembrane protein 30A; KO, knockout; CER, chimeric engulfment receptor; IFN, interferon; TNF, tumor necrosis factor; CTV, CellTrace Violet.

Transmembrane protein 30A (TMEM30A), also known as CDC50A or C6orf67, is a flippase chaperone protein that complexes with the flippase adenosine triphosphate (ATP) 11C to translocate PS and PE from the outer to the inner leaflet of the plasma membrane.^48^ We targeted *tmem30A* with CRISPR-Cas9 in the mantle cell lymphoma (MCL) cell line JeKo-1 to develop genetically modified knockout cell lines that constitutively externalize cell surface PS (TMEM30A KO). Among hematologic tumors, loss-of-function mutations in TMEM30A have been identified in approximately 5%–11% of patients with DLBCL, and among a cohort of newly diagnosed patients, this mutation was correlated with improved response to standard R-CHOP therapy.^49^ Analysis of clonal JeKo-1 TMEM30A KO cells showed stable surface PS levels of 97.3% (range: 96.7%–97.9%) (Figure 2A), thus creating a stable positive control cell line.

To evaluate cytotoxic function, T cells were co-cultured with target cells at a 1:0.5 E:T (effector to target) ratio and cytotoxicity was assessed using the IncuCyte Live-Cell Analysis System. At 120 h after co-culture, CER-1236 T cells eliminated 87% of target cells, while untransduced T cells demonstrated minimal killing (16.1%; Figures 2B and 2C). In response to co-culture with JeKo-1 TMEM30A KO cells, CER-1236 T cells secreted multiple cytokines, including IFN-γ, granzyme B, and TNF-α (Figure 2D). Cytokine secretion was dependent on binding to PS, as binding site mutant CER-1251 did not secrete cytokines in the presence of TMEM30A KO target cells. Variable levels of IL-6 and low levels of IL-10 and IL-4 were observed across all CER-1236, untransduced, and CER-1251 T cells (Figure S5).

In addition to cytotoxicity, we also evaluated the phagocytic activity of CER-1236 T cells against whole-cell targets to further assess their functionality. To accomplish this, CER-1236 T cells were co-cultured with JeKo-1 TMEM30A KO cells in the presence or absence of inhibitors of key steps of target engulfment: cytochalasin D, an actin polymerase inhibitor that prevents target uptake into phagosomes, and bafilomycin A, a lysosomal ATPase inhibitor that prevents fusion of phagosomes with lysosomes, thus preventing acidification.^50^^,^^51^ Since both agents inhibit phagocytosis and prevent the fluorescent signal increases of pHrodo Red particles that would otherwise be internalized into acidic lysosomal compartments, they would be expected to impair phagocytosis.

CER-1236 T cells demonstrated higher phagocytic activity against JeKo-1 TMEM30A KO cells than untransduced cells (Figures 2F and 2G). Phagocytic activity was reduced after treatment with either cytochalasin D or bafilomycin A in a dose-dependent manner (Figures 2F and 2G). A low dose of cytochalasin D (0.05 μM) reduced engulfment by 41%, while a dose of 0.5 μM cytochalasin D reduced CER-1236 engulfment by 79% (Figure 2F). Similarly, bafilomycin A inhibited engulfment activity by CER-1236 T cells in a dose-dependent manner, resulting in a 62% inhibition of phagocytosis at 10 nM bafilomycin A (Figure 2G). Of note, neither untransduced cells nor a TIM-4 binding site mutant CER-1250 showed engulfment of TMEM30A KO target cells.

### PS expression in human MCL and CER-1236 tumor-specific activity

**Figure 3:**
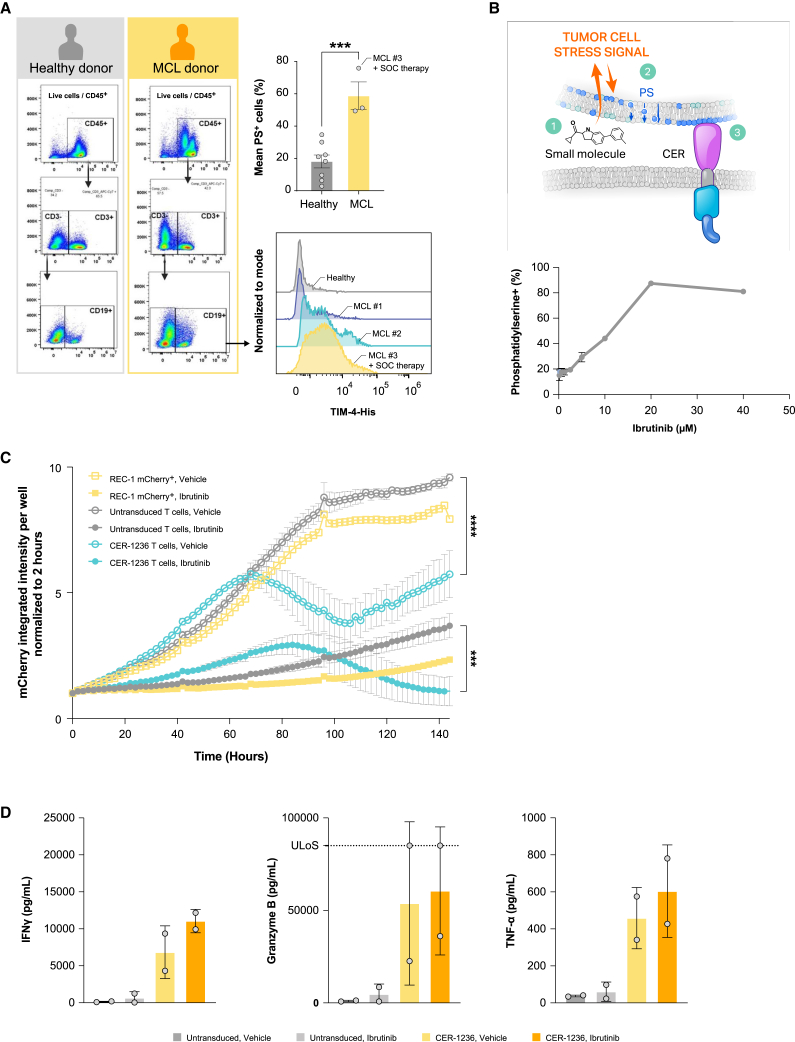
CER-1236 synergizes with BTK inhibition to eliminate mantle cell leukemia cell lines (A) Primary samples of mantle cell leukemia have high levels of PS exposure. PS exposure on PBMC samples from MCL patients or healthy donors was measured by flow cytometry. PS exposure on B cells was determined by staining with a PBMC panel and recombinant human TIM-4-His. Average ± SEM is shown (n = 8 for healthy donor, 3 for MCL patient samples). Histogram plots for MCL patients and a representative healthy donor are shown. Statistics were analyzed using an unpaired t test. ∗∗∗p < 0.001. (B) REC-1 MCL cells have constitutive exposure of PS that is increased with ibrutinib treatment. REC-1 mCherry^+^ MCL cell lines were cultured for 48 h with or without increasing concentrations of ibrutinib. PS exposure was measured on live cells by recombinant human TIM-4-His using flow cytometry. Average ± SEM is shown for technical triplicates. (C) CER-1236 synergizes with ibrutinib to eliminate REC-1 MCL cells. REC-1 mCherry^+^ cells were pretreated for 48 h with 0.5 μM ibrutinib or vehicle. REC-1 cells were washed and co-cultured with CER-1236 at a 1:4 E:T ratio in the presence or absence of 0.5 μM ibrutinib in medium supplemented with 200 IU/mL IL-2. Vehicle-treated cells and untransduced cells were included as controls. Cytotoxicity was measured by reduction in mCherry integrated intensity using the IncuCyte Live-Cell Analysis System. Average cytotoxicity ± SEM is shown (n = 3 donors). Statistics were analyzed using a two-way ANOVA with Tukey’s multiple comparisons test at each time point. Results for 144 h are reported. ∗∗∗p < 0.001, ∗∗∗∗p < 0.0001. (D) CER-1236 secrete effector cytokines in response to PS^+^ target cells. CER-1236 were co-cultured with REC-1 mCherry^+^ cells at a 1:4 ratio for 120 h in the presence or absence of ibrutinib in medium supplemented with 200 IU/mL IL-2. Untransduced cells were included as controls. After co-culture, cytokine secretion was measured by automated ELISA. Average cytokine secretion ± SEM is shown (n = 2 donors). MCL, mantle cell lymphoma; PS, phosphatidylserine; SOC, standard of care; TIM-4, T cell immunoglobulin and mucin domain containing 4; CER, chimeric engulfment receptor; IFN, interferon; TNF, tumor necrosis factor; ULoS, upper limit of sensitivity.

A variety of tumors have been shown to have increased surface PS as a result of altered plasma membrane regulation.^52^^,^^53^^,^^54^ We assessed the levels of PS exposure in primary MCL samples using FACS staining. We used the recombinant HIS-tagged extracellular domain of human TIM-4 to detect PS exposure. This reagent mimics the binding domain of CER-1236 and can specifically bind to PS.^32^ PS was reliably detected in pathologic B cells at various intensities (Figure 3A). B cells from three patients with MCL exposed PS on 49%, 51%, and 76% of cells (donors 1, 2, and 3, respectively) (Figure 3A). In comparison, healthy donors had a range of PS^+^ cells between 2.9% and 35% (Figure 3A). Representative histograms with PS detection of MCL donor-derived B cells are displayed in Figure 3A (bottom right). Both MCL donors 1 and 2 were treatment naive, while donor 3 had received multiple prior lines of therapy and was undergoing active chemo-immunotherapy at the time of sample collection, with the majority of cells PS^+^.

We next utilized REC-1, a p53 mutant MCL cell line, to evaluate *in vitro* and *in vivo* CER-1236 anti-tumor responses. Approximately 20% of REC-1 mCherry^+^ cell line cells exhibited constitutive PS expression on the cell surface, as measured by binding to soluble, recombinant human TIM-4, the extracellular receptor of CER-1236 (Figure 3B). These results were consistent with recombinant TIM-4 staining on primary MCL samples. Addition of a targeted Bruton’s tyrosine kinase (BTK) inhibitor to elicit cell damage induced further PS exposure on viable cells and enhanced TIM-4 binding, peaking at 20 μM ibrutinib treatment (87.4% ± 1.1%).

We further evaluated CER-1236-mediated anti-tumor responses in REC-1 co-culture assays. Pretreated REC-1 mCherry^+^ cells were washed and co-cultured with CER-1236 T cells or controls at an E:T of 1:4 in the presence or absence of 0.5 μM ibrutinib. We hypothesized that the increase in surface PS on viable cells would drive enhanced CER-1236 functional responses. Ibrutinib treatment of REC-1 mCherry^+^ cells led to impairment of cell growth by 120 h, although modest cell expansion occurred over the course of co-culture with ibrutinib alone (Figure 3C). In concordance with the elevated baseline PS exposure observed on REC-1 mCherry^+^ cells, CER-1236 T cells mediated a modest cytotoxic effect against REC-1 mCherry^+^ cells (Figure 3C, empty teal line). This effect is most pronounced in the first 96 h of culture with REC-1 mCherry^+^ cells in the absence of ibrutinib, but REC-1 mCherry^+^ cells expanded afterward. In contrast, CER-1236 T cells showed a robust 86.3% ± 12.9% elimination of ibrutinib-treated REC-1 mCherry^+^ cells (Figure 3C, solid teal line). Notably, minimal killing was observed by untransduced T in co-culture with REC-1 mCherry^+^ cells. In concert with the cytotoxicity data, CER-1236 T cells secreted high levels of IFN-γ, TNF-α, and granzyme B (Figure 3D) against REC-1 targets. Untransduced cells produced only low levels of these cytokines. The addition of ibrutinib led to trends in increased cytokine production from CER-1236 T cells; however, these did not reach statistical significance.

Taken together these results demonstrate that CER-1236 cells mediate both phagocytic and cytotoxic functions against MCL cell lines, which could be further augmented by increasing target ligand through BTK inhibition.

### *In vivo* CER-1236 anti-tumor responses, pharmacokinetics, biodistribution, and toxicity measurements

**Figure 4:**
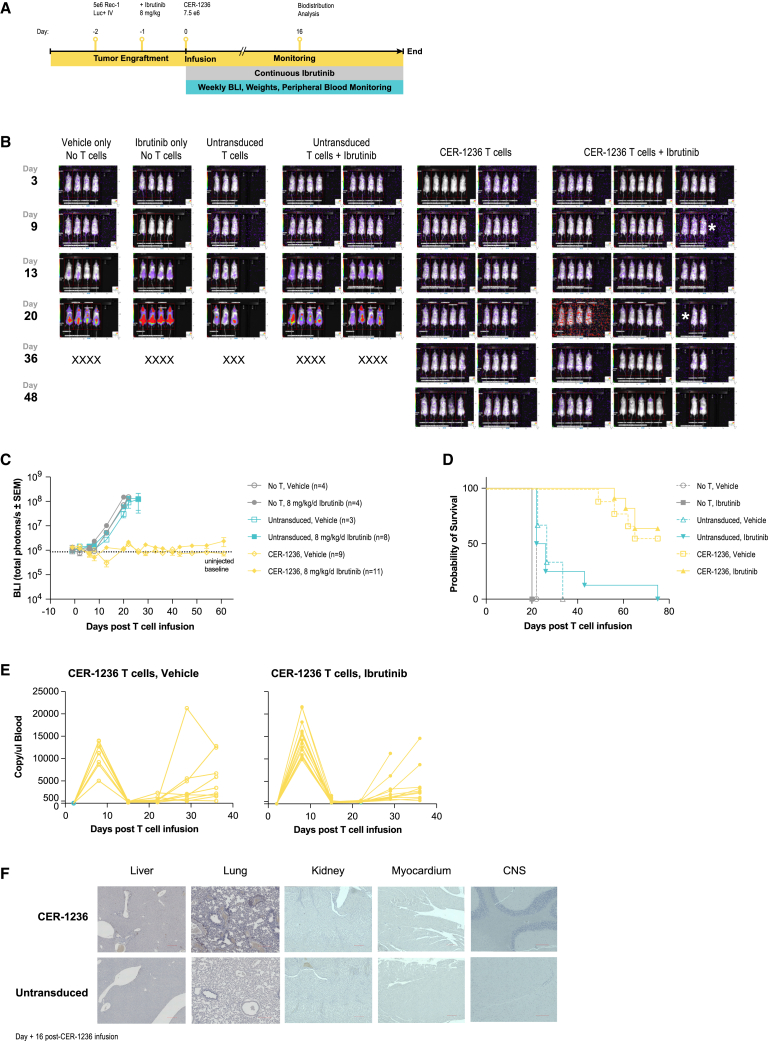
CER-1236 has potent anti-tumor effects against a xenograft model of REC-1 MCL (A) Schematic of xenograft study. 5e-6 REC-1 stably expressing fLuc were injected intravenously into NSG mice at day 2. On day 1, mice were administered 8 mg/kg/day of ibrutinib via their drinking water. Mice were administered 7.5e-6 CER-1236^+^ T cells i.v. on day 0. Tumor burden was assessed twice per week by BLI measurement, and blood was drawn weekly to assess CER-1236 T cell expansion. At day 16, organs were collected from ibrutinib-treated mice for IHC and H&E staining. (B) CER-1236 treatment eliminates REC-1 tumors. BLI images of mice taken at the indicated days post T cell transfusion. X indicates animals removed due to reaching BLI maximum, ∗ indicates animals removed for timed takedown. (C) Average BLI ± SEM shown for each group. N for each group indicated in legend. Data are representative of two experiments using different donors. (D) CER-1236 treatment improves survival in NSG mice with REC-1 MCL xenografts. Probability of survival shown for mice treated with CER-1236 or control groups. Mice were removed from study upon reaching BLI maximum of 1e-8 photons/s or upon displaying signs of GvHD. Data are representative of two experiments using different donors. (E) CER-1236 expansion peaks at 7 days post administration and decreases after. DNA was isolated from peripheral blood and frequency of CER-1236 T cells was determined by ddPCR using a CER-specific primer/probe set. Cells/μL of blood was determined by: probe counts × (volume of ddPCR reaction/μL DNA used). Longitudinal analysis is shown for each mouse in the indicated groups. (F) Anti-human CD3 IHC staining of select organs. At day 16 post T cell transfusion, untransduced CER-1236-treated animals receiving ibrutinib were sacrificed and organs were removed for IHC. Representative images of 20× images of liver, lung, kidney, myocardium, and brain are shown. Brown areas indicate CD3 detection. Scale bars, 200 μm. i.v., intravenous; CER, chimeric engulfment receptor; UNT, untransduced; SEM, standard error of the mean; BLI, bioluminescence imaging.

To assess the *in vivo* anti-tumor activity of CER-1236 T cells, immune-deficient NSG mice were xenografted with the human REC-1 cell line engineered to stably express firefly luciferase (FLuc) for noninvasive tumor measurement, then treated with 8 mg/kg/day ibrutinib or vehicle and administered CER-1236 T cells. An overview of the experiment is provided in Figure 4A. Treatment with 7.5e-6 CER-1236 T cells in the presence of ibrutinib resulted in elimination of REC-1 tumor burden in 11/11 (100%) of mice, as measured by bioluminescence imaging (BLI) (Figures 4B and 4C). CER-1236 T cells also eliminated tumors in the absence of ibrutinib treatment in 9/9 animals (100%). No tumor growth inhibition was observed in either vehicle-treated or ibrutinib-treated control groups (Figure 4C), and 16 of 19 control animals were euthanized by day 26 because of high tumor burden. Median survival for mice receiving CER-1236 T cells in the presence or absence of ibrutinib was not reached during the study period (Figure 4D).

Because PS in mouse and humans is biochemically identical, the CER-1236 extracellular component, human TIM-4, cross-reacts with mouse PS and provides insight into potential safety aspects of CER-1236 T cells. To track CER-1236 T cell pharmacokinetics *in vivo*, total CER-1236 T cells present in the blood was determined using ddPCR using probes designed against the unique CER-1236 TIM-4 and CD28 transmembrane domain junction, and control probes were designed against a region of the histone acetyltransferase (HAT) gene conserved in mice and humans. CER-1236 levels in the peripheral blood show robust expansion at 7 days in the presence or absence of ibrutinib. In mice that received CER-1236, animals showed 423.4- and 489-fold increases compared with day 2 in the absence or presence of ibrutinib, respectively (Figure 4E). High levels of CER-1236 did not persist in the periphery, and groups that received CER-1236 T cells showed an over 95% contraction at day 14 compared with the day 7 peak. Of note, a subset of animals showed evidence of late T cell activation that preceded visible signs of graft-versus-host disease (GvHD). To mitigate the effects of xenogenic GvHD, we repeated this experiment with an identical study design, donor, and production run in the NSG-(KbDb)null (IA)null mouse line, which lacks expression of class I and class II MHC and have reduced incidence of GvHD (Figure S6A).^55^ In this background CER-1236 treatment had no effect on animal body weight (Figure S6B), mediated similar anti-tumor effect as in NSG mice (Figure S6C), even at lower doses (Figures S7A and S7B), and improved mouse survival (Figures S6D and S7C). CER-1236 expanded and contracted with similar kinetics, but showed no late expansion in the periphery. Instead, the frequency of CER-1236 cells decreased to baseline (Figure S6E). These results suggest that the late T cell expansion we observe in the NSG mouse model is due to GvHD rather than on-target off-tumor binding, and are consistent with symptoms of GvHD observed in both untransduced and CER-1236-treated NSG animals.

Complete blood count, prothrombin time, and partial thromboplastin time were measured in blood taken at the peak of T cell expansion (day 7) and after T cell contraction (day 14). Hematologic indices, including hemoglobin/hematocrit, platelets, and neutrophils, remained stable throughout the study (Figure S8A), and prothrombin and partial thromboplastin time were unaffected at the peak of T cell expansion and after contraction (Figure S8B).

To assess tissue distribution of CER-1236, we isolated organs at day 16 from animals treated with untransduced or CER-1236 with 8 mg/kg/day ibrutinib and performed IHC for human CD3 and H&E staining. For these studies, CER-1236 transduction was >90%. CD3^+^ cells were not detected in the kidney, myocardium, or brain of treated mice (Figure 4F). Low levels of CD3^+^ cells were identified in the lungs and liver of T cell-treated mice, consistent with previous reports showing that the liver and the lungs are sites of engineered T cell occupancy in models of CAR T cells or engineered chimeric TCR T cells.^56^^,^^57^^,^^58^^,^^59^ The frequency of CD3^+^ cells in these tissues was higher in CER-1236-treated animals than in untransduced animals (Figure 4F). H&E-stained tissue samples demonstrated no gross histological abnormalities in either CER-1236 or untransduced T cell-treated animals of heart, lung, liver, kidney, and brain (Figure S9).

Taken together, these studies indicate that CER-1236 alone displayed robust expansion *in vivo* and had potent anti-tumor effects against REC-1 targets in the presence or absence of ibrutinib, likely a result of baseline cell surface PS observed on REC-1 cells. CER-1236 showed a restricted pattern of tissue distribution in line with other T cell products and did not result in tissue damage despite murine and human PS being biochemically identical. A trend toward increased peak expansion of CER-1236 was observed in the presence of ibrutinib *in vivo*; however, this did not reach statistical significance.

### Conditional activation of CER-1236 drives anti-tumor solid tumor responses in pre-clinical NSCLC models

**Figure 5:**
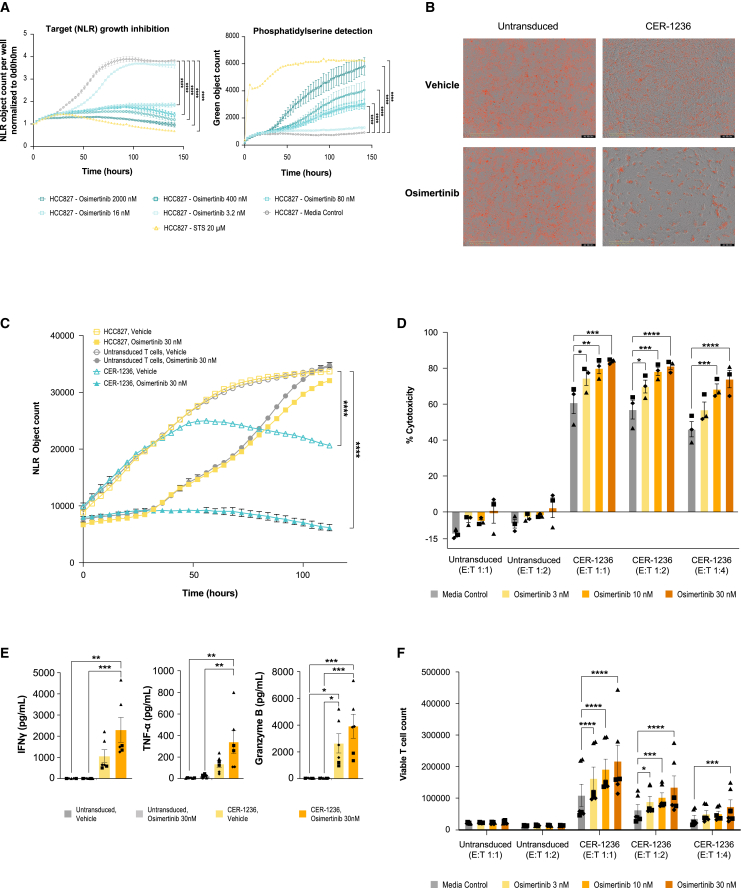
CER-1236 mediates cytotoxicity, cytokine secretion, and proliferation against EGFR mutation-positive NSCLC HCC827 cells, augmented by osimertinib, an EGFR inhibitor (A) Osimertinib treatment led to dose-dependent inhibition of HCC827 target growth expansion and increase in exposure of PS (CER-T ligand) compared with medium control. PS exposure was measured by IncuCyte real-time quantitative microscopic detection of binding of recombinant human TIM-4 ECD to externalized PS. STS included as a positive control for maximal PS induction. Average object count ± SEM shown for two technical duplicates from one representative experiment (n = 3). Statistics were analyzed using a two-way ANOVA with Dunnett’s multiple comparisons test at each time point. Results for 141 h are reported. ∗∗∗∗p < 0.0001. (B) Representative IncuCyte image overlays of phase/contrast and red channel taken at 120 h post co-culture demonstrating HCC827 target abundance or lack thereof from co-cultures. (C and D) CER-1236 co-cultures demonstrates enhanced tumor elimination in combination with osimertinib treatment. Statistics were analyzed using a two-way ANOVA with Dunnett’s multiple comparisons test at each time point and results for 112 h are reported. ∗∗∗∗p < 0.0001. CER-1236 were co-cultured with HCC827 targets at an E:T ratio of 1:1, 1:2, and 1:4 for 120 h. Vehicle-treated HCC827 targets and untransduced T cells were included as controls. Percent cytotoxicity is reported as percent change in HCC827 average target cell count from T cell co-cultures at 120 h relative to HCC827 target alone average count for the respective control or osimertinib concentrations. Percent change in average object count shown (n = 3 donors). Statistics were analyzed using a two-way ANOVA with Dunnett’s multiple comparisons. ∗p < 0.05, ∗∗p < 0.01, ∗∗∗p < 0.001 ∗∗∗∗p < 0.0001. (E) CER-1236 T cells secrete effector cytokines in response to HCC827 target cells. Co-culture with osimertinib-treated HCC827 targets led to further enhancement of secretion of IFN-γ, TNF-α, and granzyme B compared with untreated targets. CER-1236 were co-cultured with HCC827 targets at a 1:4 E:T ratio for 120 h. Vehicle-treated HCC827 targets and untransduced T cells were included as controls. After co-culture, cytokine secretion was measured by automated ELISA. Average cytokine secretion ± SEM is shown (n = 3 donors). Statistics were analyzed using one-way ANOVA with Tukey’s multiple comparisons test. ∗p < 0.05, ∗∗p < 0.01, ∗∗∗p < 0.001. (F) Enhanced proliferation of CER-1236 T cells co-cultured with osimertinib-treated targets compared with untreated target co-cultures of CER-1236 and untransduced T cell co-cultures. Proliferation was measured by flow cytometry using precision count beads to determine the absolute counts of viable T cells at the end of the 120-h co-culture. T cell proliferation is reported as viable T cell count determined. Average viable T cell count ± SEM shown (n = 3 donors). Statistics were analyzed using a two-way ANOVA with Dunnett’s multiple comparisons test. ∗p < 0.05, ∗∗∗p < 0.001 ∗∗∗∗p < 0.0001. Donors designated as follows: (▲) donor 1, (▪) donor 2, (◆) donor 3. PS, phosphatidylserine; ECD, extracellular domain; STS, staurosporine; CER, chimeric engulfment receptor; IFN-γ, interferon gamma; TNF-α, tumor necrosis factor alpha; SEM, standard error of the mean; E:T, effector to target ratio; NLR, nuclear localized red.

Because solid tumors tend to have a large degree of antigen heterogeneity, finding suitable, highly expressed cell surface tumor antigens for engineered T cell therapies to target has been a greater challenge than for hematologic tumors.^60^ EGFR mutation-positive advanced NSCLC accounts for 10%–15% of lung adenocarcinomas among Westerners and even higher among Asians.^61^ Front line EGFR tyrosine kinase inhibitors (TKIs) have been shown to have superior therapeutic effect compared with chemotherapy; nevertheless, resistance to EGFR TKIs is inevitable with a median overall survival of 38.6 months.^62^^,^^63^ We tested TIM-4 binding to EGFR mutation^+^ NSCLC cell lines, HCC827 and H1975, which harbor L858R double mutations and a 5 amino acid exon 19 deletion, respectively. Similar to our findings in MCL cell lines and primary samples, a large population of H1975 cells bound to TIM-4. This could be further increased with EGFR inhibition (osimertinib), with nearly all cells becoming PS^+^, with limited alterations in cell viability (Figures S10A and S10B), reflecting cell injury. HCC827 cells also demonstrated increases in PS upon EGFR inhibitor therapy, with nearly all cells becoming PS^+^ in culture by 100 h (Figures 5A and S10B).

The addition of CER-1236 at a low E:T ratio had a moderate killing effect against HCC827 cells (Figures 5B and 5C). However, the addition of osimertinib, the preferred EGFR inhibitor option for first-line treatment for EGFR mutation-positive advanced NSCLC, to the culture markedly enhanced CER-1236 T cell killing (Figures 5C and 5D), in line with the increases in target ligand.^64^ Percent cytotoxicities of 69.78%, 78.25%, and 81.22% were observed at 3, 10, and 30 nM osimertinib, respectively (Figure 5D). In contrast, HCC827 target cells incubated with untransduced T cells showed minimal changes in cell numbers compared with incubation in the absence of T cells (6.60%–2.32% increases) at all drug concentrations tested (Figure 5C).

Conditional cytokine and proliferation responses were also observed upon drug treatment. Supernatants from CER-1236-treated target cells had IFN-γ levels over 400-fold higher than that of untransduced T cell target cell co-cultures. Addition of osimertinib to co-cultures further increased IFN-γ levels by more than 2-fold compared with CER-1236 treatment alone (Figure 5E). Similar trends were observed with TNF-α and granzyme B (Figure 5E). Consistent with the observed cytotoxicity and cytokine responses, rapid expansion of CER-1236 was detected in co-culture, with the greatest CER-1236 T cell expansion observed with drug treatment (Figure 5F). Increases in osimertinib concentrations led to dose-dependent CER-1236 proliferation. In contrast, untransduced T cells demonstrated minimal proliferation, with no observed drug-dependent proliferation. CER-1236 also exhibited enhanced cytotoxicity, cytokine secretion, and proliferation in co-culture with H1975 targets (Figures S10C–S10E). Taken together, these results demonstrate CER-1236 anti-tumor function against EGFR mutation-positive NSCLC. Furthermore, effector responses could be markedly enhanced by upregulating target ligand using a standard of care EGFR inhibitor.

### Tumor clearance of NSCLC adenocarcinomas after CER-1236 infusion

**Figure 6:**
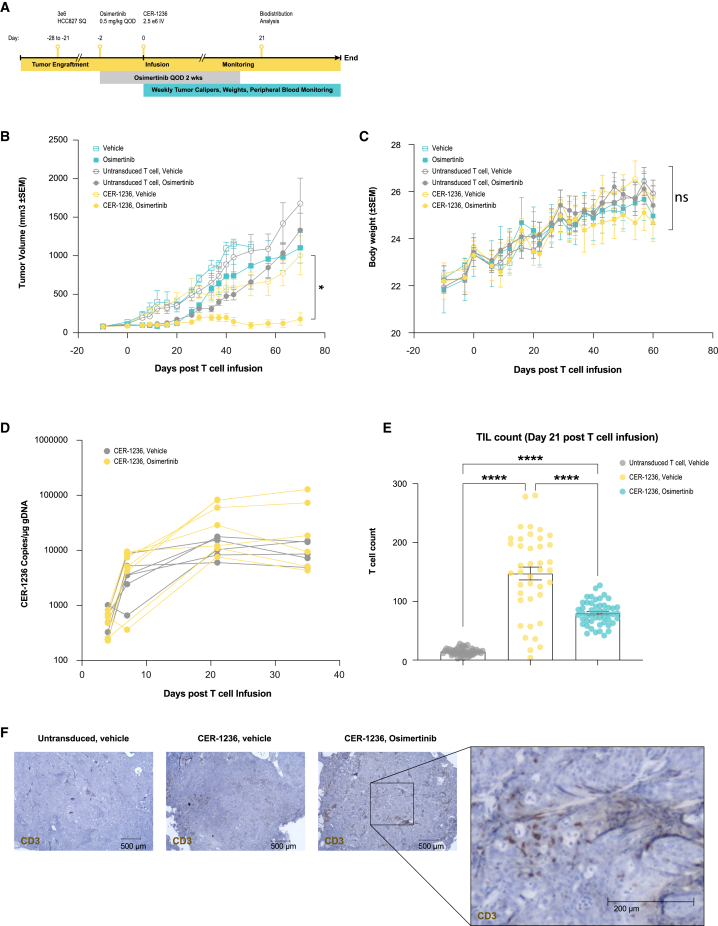
CER-1236 *in vivo* efficacy against EGFR mutation-positive NSCLC xenografts (A) Schematic of schedule of assessments for evaluation of CER-1236 anti-tumor activity against a localized xenografted NSCLC (HCC827) solid tumor model in NSG mice with five to eight animals per group. (B) CER-1236-treated animals led to NSCLC xenograft tumor regression, with combination of osimertinib (0.5 mg/kg, QOD for 14 days) and CER-1236 leading to the largest regression in tumor volume. Average tumor volume ± SEM. Statistics were analyzed at each time point using a mixed-effects analysis with the Geisser-Greenhouse correction with Tukey’s multiple comparisons test. Results are reported for day 70. ∗p < 0.05. (C) Osimertinib or T cell treatments did not lead to observable loss in body weight. Average body weight ± SEM. (D) CER-1236 infused to osimertinib-dosed HCC827 animals showed higher CER T cell expansion compared with CER-1236 infused in vehicle-treated controls after a single infusion of 2.5e-6 T cells. CER-1236 T cell expansion in blood was evaluated by ddPCR analysis using CER-1236-specific probes on DNA isolated from check bleed samples. Statistics were analyzed at each time point using a mixed-effects analysis with the Geisser-Greenhouse correction with Dunnett’s multiple comparisons test. (E and F) Higher T cell infiltrates were observed in CER-1236-treated animals from day 21 harvested tumor sections. The bar graph represents average CD3-stained automated T cell counts from 40 to 50 images taken at 20× magnification of two adjacent tissue sections from the same tumor from the described groups. Average ± SEM (n = 1). Statistics were analyzed using a one-way ANOVA with Tukey’s multiple comparisons test. ∗∗∗∗p < 0.0001. A 4× captured representative image with overlays of CD3 (brown)-stained sections from tumors harvested at day 21. NSCLC, non-small cell lung cancer; CER, chimeric engulfment receptor; OQD, every other day; SEM, standard error of the mean; ddPCR, digital droplet polymerase chain reaction.

We engrafted NSG animals with localized HCC827 cells, and assigned animals to receive a short course of EGFR inhibition to prime tumors with target antigen and CER-1236 T cells (Figure 6A). We hypothesized the simultaneous exposure to both therapies would lead to synergistic *in vivo* anti-tumor responses. HCC827 NSCLCs were inoculated into the flanks of NSG mice and, once established (∼20 days post-engraftment), tumors were infused with 2.5e-6 CER-T^+^ cells. Drug alone treatment groups (Figure 6B), after initial tumor regression, developed progressive disease. In contrast, animals infused with CER-1236 T cells demonstrated potent anti-tumor responses in the presence of osimertinib (Figure 6B). As with the MCL studies in NSG animals, no evidence of organ toxicity or weight loss (Figure 6C) was observed, with increases in body weight recorded in all groups over the course of the study. CER-1236 T cells expanded rapidly in the blood, with the highest expansion observed in the osimertinib-treated groups (Figure 6D). Gross analysis and immuno-histochemical staining of the tumors post-infusion demonstrated extensive infiltration of T cells compared with untransduced controls (Figures 6E and 6F).

### CER-1236 induction of APC-like responses

**Figure 7:**
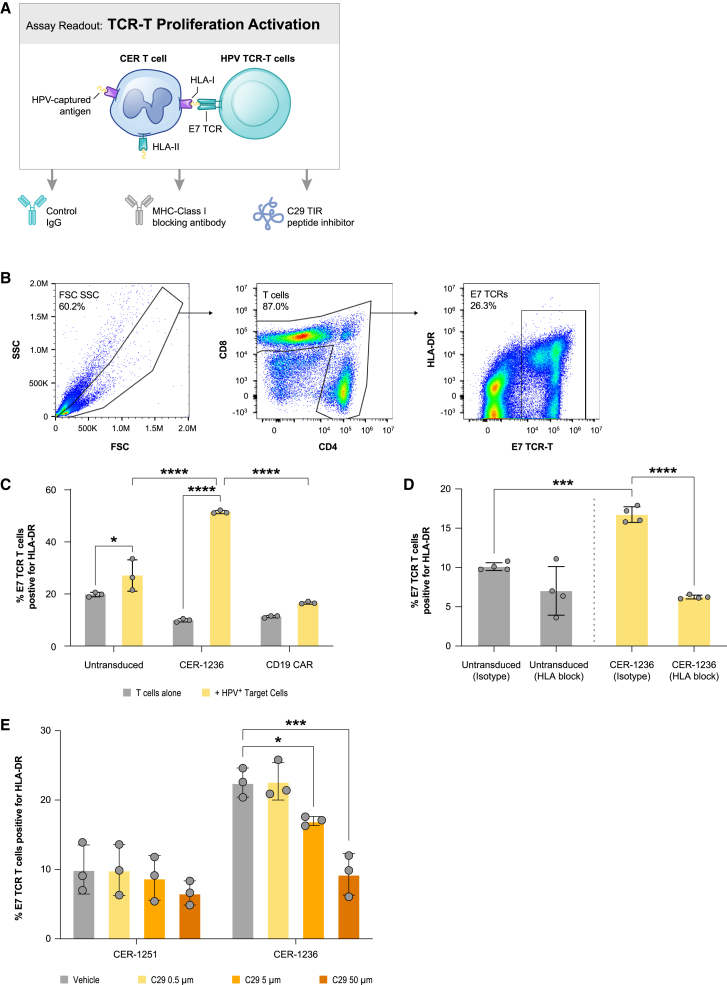
CER-1236 can cross-present E7 antigen in an HLA class I- and TLR2-dependent manner (A) Schematic of cross-presentation assay. After 48 h co-culture with E7^+^ SCC152 TMEM30A KO cells, CER-1236 cells, or control cells were isolated with CD3-positive selection and co-cultured with E7 TCR^+^ T cells at a 1:1 ratio for 96 h. Activation was measured by upregulation of the activation marker HLA-DR on E7 TCR^+^ T cells. C29 TLR2 inhibitor can be used in the first step to impair antigen uptake, and HLA class I blocking antibody can be used in the second step to impair cross-presentation. (B) Flow gating to identify HLA-DR upregulation on E7 TCR^+^ T cells. (C) CER-1236 can activate E7 TCR^+^ T cells only after co-culture with E7^+^ SCC152 TMEM30A KO cells. CER-1236, untransduced, or CD19-CAR with a CD3/28 signaling domain were co-cultured in the presence or absence of SCC152 TMEM30A KO cells for 48 h, positively selected for CD3, then co-cultured with E7 TCR^+^ T cells for 96 h. Average ± SEM of three technical replicates is shown. Representative experiment of two repeats shown for CER-1236 and untransduced cells. Statistics were analyzed using a one-way ANOVA with Tukey’s multiple comparisons test. ∗p < 0.05, ∗∗∗∗p < 0.0001. (D) CER-1236-mediated activation of E7 TCR^+^ T cells is dependent on HLA class I. CER-1236 or control T cells were co-cultured with E7 TCR^+^ T cells as in (C), in the presence or absence of 100 μg/mL HLA class I blocking antibody or isotype control added during the second step. Average ± SEM of four technical replicates is shown. Statistics were analyzed using a one-way ANOVA with Tukey’s multiple comparisons test. ∗p < 0.05, ∗∗∗p < 0.001, ∗∗∗∗p < 0.0001. (E) TLR2 signaling contributes to CER-1236 activation of E7 TCR^+^ T cells. CER-1236 or control T cells were co-cultured with SCC152 TMEM30A KO cells in the presence of varying concentrations of the TLR2 inhibitor C29, then co-cultured with E7 TCR^+^ T cells in the presence of varying concentrations of C29. The average ± SEM of three technical replicates is shown. Statistics were analyzed using a one-way ANOVA with Dunnett’s multiple comparisons test within each construct. ∗p < 0.05, ∗∗∗p < 0.001.

It has previously been described that T cells have the capacity to present antigen, but have inefficient mechanisms for antigen uptake.^38^^,^^39^ We sought to determine if the phagocytic capacity we engineered into CER-1236 T cells improved the ability of T cells to present target antigen. The immunodeficient animal models utilized above lack the ability to fully model host immune responses after CER-1236 T cell infusion and we therefore utilized *in vitro* models of antigen presentation to evaluate CER-1236 T-APC responses. We developed a two-step assay in which CER-1236 T cells were co-cultured for 48 h with the HPV^+^ SCC152 squamous cell carcinoma cell line engineered to be constitutively PS^+^ by TMEM30A KO. SCC152 cells also constitutively express the HPV protein E7. Co-culture with CER-1236 led to killing of target cells. Subsequently, CER-1236 T cells were purified from bulk cultures using CD3 isolation, then co-cultured with autologous T cells engineered to express the E7-specific TCR (Figure S11), to evaluate whether CER-1236 could trigger E7-TCR-T-specific responses.^65^ Activation of the E7 T cells was measured by upregulation of HLA-DR, a marker of T cell activation. (Figure 7A). Co-culture of E7 TCR T cells with either untransduced T cells or CD19-CAR-transduced control T cells, with or without preexposure to SCC152 TMEM30A KO cells, or with CER-1236 T cells not pre-exposed to SCC152 TMEM30A KO cells, did not affect E7 TCR T cell activation. However, co-culture of E7 TCR T cells with CER-1236 T cells pre-exposed to SCC152 TMEM30A KO cells led to increased activation (Figures 7B and 7C). Because the E7 TCR is dependent on antigen exposure in class I HLA-A2, we tested if this phenomenon could occur in the presence of HLA class I blockade. HLA-DR expression on E7 TCR T cells was reduced to baseline in the presence of HLA class I blocking antibodies (Figure 7D), indicating that the interaction between CER-1236 and E7 TCR T cells that leads to activation is dependent on HLA class I.

Next, we sought to determine if the ability of CER-1236 to uptake antigen was dependent on signaling through the TLR2 domain in CER-1236 during the co-culture with SCC152 TMEM30A KO cells. We added C29, a small-molecule inhibitor of TLR2 signaling, to the co-culture of SCC152 TMEM30A KO cells and CER-1236 T cells. This resulted in a dose-dependent decrease of activation of E7 TCR T cells, indicating a role for TLR2 signaling in the ability of CER-1236 T cells to cross-present antigen (Figure 7E). Together these data indicate that CER-1236 T cells that have been activated with an antigen-expressing target cell can acquire that antigen in a TLR2-dependent manner and cross-present antigen to third-party T cells via class I HLA.

## Discussion

Autologous adoptive immunotherapy has made significant progress in recent years.^66^^,^^67^ While these therapies have shown potential for curing patients, they are not without limitations. For patients who do respond, analysis of tumor biopsies and TCR profiles suggest that the engagement of the endogenous host response, including bystander lymphocytes and TCR clones directed against non-targeted tumor-associated antigens, may be critical for achieving clinical success.^68^^,^^69^^,^^70^

We have developed a novel cellular immunotherapy platform that activates a collaborative innate-adaptive anti-tumor immune response through the recognition of specific exposure of PS on tumor surfaces. Our approach employs T cell activating domains CD3ζ and CD28, which promote potent T cell activation and cytotoxicity against PS-bearing cells, while TIM-4 confers CER-1236 T cells with pro-phagocytic function. The acquisition of tumor antigen to CER-1236-activated T cells is accompanied by enhanced CER-1236 cross-presentation, as evidenced by the triggering of E7-specific TCR-T responses in a class I and TLR-2-dependent manner. The fact that CER-1236 can selectively present tumor-associated antigen could therefore have immunologic consequences in a clinical setting. Because antigen heterogeneity and immunoediting by tumor cells is one mechanism for escape that helps limit T cell attack, the possibility exists to broaden host anti-tumor responses to non-targeted antigens by CER-1236 T cells. In this model, CER-1236 not only mobilize proinflammatory (IFN-γ, TNF-α, chemokines) and cytotoxic functions but also process tumor-associated antigens to generate tumor antigen-specific T cells, and therefore overcome antigen presentation defects in the tumor microenvironment.

Our findings also underscore the potential multi-dimensional therapeutic utility of targeting the membrane phospholipid, PS. A variety of tumors have been shown to have increased cell surface PS, which can act as a signal for phagocytosis due to plasma membrane dysregulation.^52^^,^^53^^,^^54^ Our studies on lymphoma and EGFR mutation-positive NSCLC cell lines and primary samples have further confirmed the upregulation of PS on viable tumor cells. The addition of targeted therapeutics to further increase target antigen density has been shown to augment CER-1236 functional responses, resulting in greater cytokine secretion, T cell proliferation, and cytotoxicity. However, while precision medicine inhibitors have demonstrated clinical successes, they often lead to partial responses and relapse. These results provide a rationale for using CER-1236 T cells to augment the efficacy of targeted inhibitor therapies. Importantly, our data also indicate that the combination of CER-1236 with either a BTK or EGFR inhibitor did not result in toxicity to normal tissues, nor does it affect peripheral blood counts or clotting kinetics, indicating the selectivity of the anti-tumor immune response. This selectivity is crucial and reflects how the regulation of eat me signals in normal tissue homeostasis is tightly regulated to prevent unwanted phagocytosis of healthy cells.

Overall, the results reported here provide support for initiating clinical translation efforts to develop CER-1236 T cell therapy. Targeting multiple tumor antigens, identifying optimal combinations with other therapies, and testing CER-1236 T cell combinations with other immunotherapeutic approaches appear warranted.

## Materials and methods

### Generation of lentiviral vectors

Replication-defective lentiviruses were produced via standard methods using third-generation lentiviral plasmid encoding the extracellular domain of TIM-4, CD28 transmembrane domain, and CD28, CD3ζ, and TLR2/TIR-1 intracellular signaling domains.^71^ The lentiviral plasmid was mixed with three packaging plasmids encoding VSV-G (Aldevron), gag/pol (Aldevron), and rev (Aldevron), then transfected into HEK293T cells (ATCC) using polyethyleneimine linear (Polysciences). A lentiviral plasmid encoding a mutated extracellular domain of TIM-4 to prevent PS binding and the same transmembrane and intracellular domains listed above, was used to generate a control vector. In some experiments, pHR plasmid encoding GLuc4-mCherry3xNLS under the control of hEF-1α promoter (Elim Biopharm) was also used to generate an mCherry-LV vector.

### Generation of CER-1236 and E7 TCR T cells at small scale

Healthy donor T cells were positively selected from fresh leukopak provided by AllCells (Alameda, CA) by using anti-CD4 and CD8 antibodies through the Prodigy TCT program. All related reagents and instruments were purchased from Miltenyi. After selection, T cells were either cryopreserved or activated in optimizer medium by using TransAct in an NTC 6-well culture plate. After activation, cells were re-plated in a 24-well non-tissue culture-treated plate in fresh culture medium, transduced with lentivirus encoding CER-1236 or a control construct, CER-1251, which contains a mutation that renders the TIM-4 receptor unable to bind to the PS target and has the same signaling domains as CER-1236. E7 TCR T cells were transduced with a lentivirus encoding the E7 TCR and a truncated EGFR.^65^ After transduction, cells were transferred to a 6-well G-Rex dish and 39 mL of fresh OpTmizer T cell culture medium was added. Seventy-five percent of the medium was refreshed during the culture. On harvest day, 20 mL of medium was removed, cells were resuspended in their remaining 20 mL medium, counted, and centrifuged at 400 × *g*. Following removal of the supernatant, the cell pellet was resuspended in appropriate medium or buffer for later assays or cryopreservation.

### Generation of CER-1236 cells at large scale

T cells were selected as indicated above. After selection, a fixed amount of cells were used to start the TCT program on Prodigy. Cells were cultured in Optimizer medium, and activated by using GMP grade TransAct. After activation, cells were transduced with lentivirus encoding CER-1236 in the presence of TransAct. After transduction, cells were washed and resuspended in fresh medium for expansion. During the expansion, medium was supplied or refreshed. At harvest day, cells were harvested into freshly prepared optimizer medium. After cell counting, cells were distributed for different assays or cryopreservation. The entire process happened in the culture chamber of a TS520 tubing set, and was controlled using a TCT program.

### Assessment of transduction efficiency

As a measure of transduction efficiency, transduced CER-1236 cells were stained and analyzed by flow cytometry for detection of TIM-4, which is not expressed on normal human T cells. Transduced E7 TCR T cells were stained and analyzed for detection of the murine TCRβ, a component of the E7 TCR constant region. In brief, at the end of cell production, cells were washed, resuspended in a 1:1 mixture of autoMACS running buffer (Miltenyi Biotec) and Brilliant stain buffer (BD Biosciences, Franklin Lakes, NJ) and incubated for 30 min on ice with Fixable LIVE/DEAD viability dye (Invitrogen, Waltham, MA), anti-CD3 (clone SK7, BioLegend, San Diego, CA), and mouse anti-human TIM-4 (clone 921832, R&D Systems, Minneapolis, MN) or hamster anti-mouse TCRβ (clone H57-597, BioLegend). Cells were washed in autoMACS running buffer, then resuspended and acquired on a CytoFLEX LX (Beckman Coulter). The presence of TIM-4 or murine TCRβ on the cell surface was assessed on live CD3^+^ cells to determine transduction efficiency of CER-1236 or E7 TCR T cells, respectively; gating was set using fluorescence minus one (FMO) controls for each donor.

### Measurement of target-dependent IFN-γ secretion by CER-1236 T cells

To generate plates coated with PS, porcine brain-derived PS (Avanti Polar Lipids, Birmingham, AL) or PE (Avanti Polar Lipids) were dissolved in methanol to a 50 μg/mL stock. Two-fold serial dilutions of this PS stock were generated in methanol, corresponding to final concentrations of 25, 12.5, 6.25, 3.13, 1.56, 0.78, 0.39, and 0 μg/mL. The 0 μg/mL wells are treated with methanol and allowed to evaporate to serve as the uncoated unstimulated control. Each stock (20 μL) was added to a flat-bottomed 96-well plate and rocked to ensure uniform coverage of the surface, then allowed to evaporate with the lid off in a biosafety cabinet.

Cryopreserved CER-1236 T cells, CER-1251 binding site mutant (control) T cells, or untransduced (control) T cells prepared as described above were thawed into 9 mL of pre-warmed OpTmizer T cell expansion medium were centrifuged at 400 × *g* for 5 min at room temperature, then the supernatant was removed and cells were resuspended in 5 mL of fresh medium. Cells were counted on a Beckman Coulter Vi-Cell BLU cell counter (Beckman Coulter, Indianapolis, IN). Cells were resuspended to a final concentration of 0.125e-6 cells/mL in medium. T cells (25,000) in 200 μL of medium were added to 2 wells coated with each PS dilution to assess target-dependent secretion of IFN-γ, as well as the single PE dilution and the unstimulated control wells to assess background IFN-γ secretion by T cells, and then incubated for 24 h at 37°C in 5% CO_2_. After incubation for 24 h, supernatants were collected and transferred to a 96-well U-bottomed plate and centrifuged for 5 min at 400 × *g* to pellet any cells. Supernatant was then transferred to a new 96-well U-bottomed plate and frozen at −80°C. Secretion of IFN-γ was measured using the ProteinSimple Ella automated immunoassay system. In brief, frozen supernatants were thawed and diluted 1:10 in Ella sample diluent (Bio-Techne, Minneapolis, MN). Samples were loaded into an 1x72 Ella IFN-γ cartridge. Duplicates were measured independently and averaged.

### Bead-based phagocytosis assay

PS-coated agarose beads of 0.5 μm diameter (Echelon Biosciences, Salt Lake City, UT) were washed with PBS containing 0.5% Tween 20 and centrifuged at 2,500 × *g* and room temperature for 5 min. This was followed by two washes with PBS containing 100 U/mL penicillin/streptomycin. The beads were resuspended with 1 mL of medium, sonicated in a water bath at room temperature for 5 min, and diluted with OpTmizer medium. Diluted beads were added to untreated 96-well flat-bottomed plates at 5,000 beads to yield a cell-to-bead ratio of 5:1. Thawed or fresh CER-1236, CER-1251, or untransduced T cells were counted and diluted with OpTmizer medium, and 25,000 live cells were added to the bead-containing plates. Samples were tested in triplicate per condition. Plates were incubated in a humidified 37°C chamber containing 5.0% CO_2_ for 16 h. To measure engulfment following cell incubation, samples of CER T cells and beads were stained with 1:100 anti-human CD3-fluorescein isothiocyanate (BioLegend, San Diego, CA) and 1:1,000 fixable viability dye eFluor 780 (Thermo Fisher Scientific) on ice for 20 min. After staining, cells were washed with pH 8 phosphate wash buffer (Fisher Scientific, Pittsburgh, PA). The pH 8 phosphate buffer was used to test the effect of quenching the pHrodo signal of extracellular beads that could be tethered to cell surfaces. Samples were acquired on the CytoFLEX Flow Cytometer (Beckman Coulter, Brea, CA). Parallel samples were imaged at 40× magnification using a Keyence BZ-X710 microscope (Osaka, Japan).

### Cell-based phagocytosis assay

Prior to co-culture, target cells were labeled with pHrodo Red. JeKo-1 TMEM30A KO cells were washed, incubated with 0.2 mg/mL pHrodo Red dye for 30 min at room temperature and washed with RPMI medium containing 20% FBS buffered with 20 mM HEPES. The stained cells were resuspended with medium. Cells were recounted and resuspended.

CER T cells were counted, washed, and stained at 1e-6 cells/mL of PBS containing 2.5 mM CellTrace Violet (Thermo Fisher Scientific, San Jose, CA) for 20 min at 37°C. Cells were then washed with RPMI medium containing 20% FBS, recounted, and resuspended with medium. T cells were plated at a 1:2 ratio into non-tissue culture-treated flat-bottomed 96-well plates.

When used, dimethyl sulfoxide stocks of bafilomycin A1 or cytochalasin D (both from Thermo Fisher Scientific) were added to the CER T cell suspensions at the initiation of co-culture. Plates were incubated for 40 h, then stained for flow cytometry and microscopy as described for bead-based phagocytosis. To determine percent phagocytosis, cells were gated on CTV to identify T cells, then gated for pHrodo Red^+^ events to identify T cells that phagocytosed target cells. Samples were run in quadruplicate.

### Generation of JeKo-1 mCherry cells

JeKo-1 cells (ATCC) were stably transduced with the mCherry gene so that these cells could be tracked in functional assays. Transduction was performed with mCherry-LV vector at an MOI of 1 in the presence of 4 μg/mL polybrene (MilliporeSigma, Burlington, MA) and a transduction efficiency of 54.1% was achieved as determined by flow cytometry. Transduced cells were sorted on a Sony SH800S cell sorter to yield a 92.9% mCherry-enriched population (termed JeKo-1 mCherry cells), which was used for further gene KO experiments.

### Cell electroporation with Cas9 ribonucleoproteins

The CRISPR-Cas 9 system was used for gene editing of JeKo-1 mCherry cells or squamous cell carcinoma cell line (SCC152, ATCC). A protocol adapted from Xia et al. was used to electroporate these cells.^72^ sgRNAs obtained from Synthego (Redwood City, CA) targeting the TMEM30A gene were resuspended in EDTA buffer (Thermo Fisher Scientific) and mixed at a 1:1:1 ratio. The sgRNA pool was mixed with Cas9 protein (Thermo Fisher Scientific) and then incubated for 10 min at room temperature to form the ribonucleoprotein (RNP) complex. After incubation, 250,000 cells, previously washed in PBS and resuspended in buffer R (Thermo Fisher Scientific), were added to the RNP mixture. The cell suspension was electroporated using a Neon system (Thermo Fisher Scientific) at 1,650 V, 13 ms, and 2 pulses. After electroporation (EP), cells were cultured following the manufacturer’s instructions.

### Evaluation of KO efficiency

One week after EP, engineered cells were evaluated for KO efficiency comparing non-EP versus EP cells. The following parameters were considered for this evaluation: (1) baseline expression of PS via flow cytometry and (2) insertion-deletion (indel) formation assessment via Sanger sequencing. In the specific case of indel formation analysis, genomic DNAs were extracted using QuickExtract Solution (Lucigen, Middleton, WI) and then submitted to Elim Biofarm for Sanger sequencing using the following primers: set 1, forward: AGAAACAGAGGGAAGGGGG; reverse: TTCCTGTCAGGGTCAGCCG; set 2, forward: GCAAATTCAGGAAACCCGCA; reverse: GTTCCTGTCAGGGTCAGCC. Sequencing data were then analyzed using the ICE online program (Synthego).

### Bulk RNA sequencing and analysis

For transcriptome profiling by RNA sequencing, 1e-6 1236 CER T cells, 1251-T cells, or untransduced T cells manufactured from three independent donors were seeded into a 6-well platform and cultured for 24 h with plate-bound PS or PE (negative control) at a single, optimized concentration. Cell pellets were snap-frozen in liquid nitrogen and shipped on dry ice to Genewiz for RNA sequencing. The RNA sequencing workflow after RNA isolation included poly(A) selection-based mRNA enrichment, mRNA fragmentation, and random priming with subsequent first- and second-strand complementary DNA synthesis. Afterward, end-repair 5′ phosphorylation and adenine nucleotide (dA)-tailing was performed. Finally, adaptor ligation, polymerase chain reaction (PCR) enrichment, and Illumina NovaSeq technology-based sequencing with 2 × 150 base pair read length were carried out. BAM files from bulk RNA sequencing were converted to merged FASTQ files. Reads were mapped to the UCSC hg19 human transcriptome using Bowtie and transcripts per million values were calculated. Samples passed quality control if the number of aligned reads was greater than 107, the percent of reads mapped was above 30%, and the percent of rRNA was in the range of 10%–30%. Genes with an adjusted p value (Padj) of <0.05 were referred to as differentially expressed genes. IPA analysis was performed by analysis of the respective DEGs. Raw and processed data were uploaded to GEO (accession no. GSE232108).

### Measurement of PS induction on REC-1 MCL cells

A 10 mg/mL stock solution of ibrutinib (MedKoo Biosciences, Morrisville, NC) was formulated in 20% 2-hydroxypropyl-b-cyclodextran (Sigma-Aldrich, St. Louis, MO). Serial dilutions of ibrutinib were prepared using RPMI medium and added to cells. Vehicle-treated samples received the equivalent vehicle concentration at the highest dose of ibrutinib (0.07%). Plates were incubated in a humidified 37°C chamber containing 5.0% CO_2_ for 48 h. Cells were resuspended, washed, and stained with LIVE/DEAD Fixable Aqua viability dye, 2 mg/mL recombinant human TIM-4-His (R&D Systems), and then stained with anti-His AF647 mAb. Each incubation step occurred in an assay buffer for 30 min on ice, followed by washing with assay buffer and centrifugation at 400 × *g* and 4°C for 5 min. Stained cells were analyzed on a CytoFLEX Flow Cytometer (Beckman Coulter).

### Measurement of PS induction on NSCLC targets by IncuCyte

HCC827 mKate2 nuclear localization red-expressing target cells were plated in 96-well flat-bottomed plate in F-12K medium supplemented with 10% FBS. NSCLC targets were allowed to attach for 4 h in an incubator and then treated with the described concentrations of osimertinib (LC Labs, Woburn, MA) or staurosporine (Selleck Chemicals, Houston, TX). Phosphatidylserine detection reagent was prepared using recombinant hu-TIM4 ECD-his (R&D Systems) and His-Tag (D3I1O) XP Rabbit mAb (Alexa Fluor 488 Conjugate) (Cell Signaling, Danvers, MA), and were added to the drug-treated NSCLC targets at final concentrations of 0.62 and 0.2 μg/mL, respectively. The plate was placed in an IncuCyte Live-Cell Analysis System for 4× image acquisition in phase, red, and green channels at 4-h intervals.

### Measurement of CER T cell cytotoxicity and cytokine secretion against target cells

CER-1236 T cells were co-cultured with engineered JeKo-1 mCherry^+^ TMEM30A KO cells or tumor cell lines pretreated with small-molecule inhibitors. For co-culture with JeKo-1 mCherry^+^ TMEM30A KO cells, target cells, and T cells were co-cultured at a 1:1 ratio in medium supplemented with 200 IU/mL IL-2. For co-culture with ibrutinib-treated REC-1 mCherry^+^ cells, REC-1 cells were pretreated with 0.5 μM ibrutinib for 48 h, then washed, recounted, and co-cultured with CER-1236 T cells at a 1:4 E:T ratio in medium supplemented with 200 IU/mL IL-2 and 0.5 μM ibrutinib. Vehicle-pretreated REC-1 cells were included as controls and not treated with ibrutinib during co-culture.

HCC827 (CRL 2868, ATCC, Manassas VA) were engineered through lentiviral transduction to express nuclear localization red/mKate2 (Sartorius, Germany). HCC827 target cells were pretreated with 30 nM osimertinib in a 96-well flat-bottomed plate for 48 h. CER-1236 T cells were co-cultured with osimertinib-pretreated HCC827 targets at varying E:T ratios of 1:1, 1:2, and 1:4 in cytokine-free medium with 15 nM of continuous osimertinib during co-culture. Vehicle-treated HCC827 were included as untreated controls to evaluate osimertinib-induced CER T cell function.

Cytotoxicity was monitored using the IncuCyte Live-Cell Analysis System with images acquired every 2 h. Reduction of red mCherry^+^ signal was used to determine target cell elimination. Untransduced T cells or CER-1251 T cells, containing a TIM-4-binding site mutant, were used as negative controls. Parallel plates were prepared for supernatant collection at 120 h. Cytokine secretion was measured using the Ella automated immunoassay platform (Biotechne, Minneapolis, MN).

### Antigen presentation assays

SCC152 E7-expressing TMEM30A KO cells were trypsinized, counted, and plated for 72 h at 37°C. CER-1236 or control T cells were added to each well in the presence or absence of the TLR2 inhibitor C29 and co-cultured for 24 h at 37°C. At the end of co-culture, cells were resuspended in autoMACS Rinsing Solution (Miltenyi Biotec) and CD3^+^ cells were isolated using a CD3 Positive Selection Kit (Miltenyi Biotec). CER-1236 or control T cells were counted, then co-cultured with CTV-labeled E7 TCR T cells at a 1:1 ratio in the presence or absence of Ultra-LEAF Purified anti-human HLA-A,B,C (BioLegend) for 96 h. At the end of the co-culture, cells were harvested, washed, and stained using CD4 (BioLegend), CD8 (BioLegend), LIVE/DEAD Aqua (Thermo Fisher Scientific), HLA-DR (BioLegend), and anti-mouse TCRβ (binds to E7 TCR; BioLegend). After staining, cells were washed, then analyzed on a CytoFLEX (Beckman Coulter).

### Cryopreserved PBMC samples and immunophenotyping using flow cytometry

Patient-derived PBMC samples with MCL were obtained from Discovery Life Sciences (Huntsville, AL) under IRB and ethics committee approval. Healthy donor-derived PBMCs were isolated from LRS chambers provided by Stanford Blood Center, (Stanford, CA), or Leukopak provided by BioIVT (Westbury, NY), HemaCare (Northridge, CA), Miltenyi Biotec (San Jose, CA), and ALLCells (Alameda, CA). Leukopak samples were received on wet ice, processed for PBMC isolation, and stored in liquid nitrogen until processed. Donor information is listed in Table 1.

**Table 1:** Patient characteristics

Sample ID	Donor ID	Gender	Age (years)	Treatment status	Treatment history
MCL 1	BBX1000- N3122064314101121SH	male	50	newly diagnosed	N/A
MCL 2	BBX1000-E7120374102050415SH	male	65	treatment naive	N/A
MCL 3	BBX1000-SC120904028062321SH	male	68	BR (cycle 1, day 1)	prior lines of therapy: ibrutinib, rituximab, BR, rituximab, stem cell transplant, HiDAC-R, R-CHOP, zanubrutinib

BR, bendamustine + rituximab; HiDAC-R, high-dose cytarabine + rituximab; N/A, not applicable or not available; R-CHOP, rituximab + cyclophosphamide, doxorubicin hydrochloride (hydroxydaunorubicin).

Cryopreserved PBMC vials were thawed at 37°C and the contents of the vials were immediately transferred into cold (4°C) assay buffer prepared by adding 1% bovine serum albumin (MACS BSA Stock Solution, Miltenyi Biotec, Germany, no. 130-091-376) and 0.5 mM calcium chloride (CaCl_2_) to Dulbecco’s phosphate-buffered saline (PBS) without calcium or magnesium and maintained on wet ice. Cells were washed, centrifuged at 300 × *g* for 5 min, and resuspended in 2 mL of the ion-free PBS. Cell viability was measured using the trypan blue dye exclusion method on a Vi-CELL BLU cell viability analyzer (Beckman Coulter, Indianapolis, IN). Cells were then washed and resuspended in assay buffer. Unstained controls were prepared similarly and were prepared on the day of experiment.

To detect PS on the surface of various immune cells, cells were stained with recombinant human TIM-4 His-Tag Protein, Carrier-Free (R&D Systems, cat. no. 9407-TM-050) followed by a secondary fluorescently labeled His-Tag (D3I1O) XP Rabbit mAb (Alexa Fluor 647 Conjugate, CST, Danvers, MA, no.14931). In brief, an antibody master mix was prepared by adding primary antibody panel consisting of LIVE/DEAD Violet Dead Cell Stain Kit (Invitrogen, L34963), CD45 (clone HI30, BD Biosciences, San Jose, CA, no. 564047), CD3 (clone SK7, BD Biosciences, no. 341090), CD19 (clone HIB19, BioLegend, San Diego, CA, no. 302206), CD20 (clone 2H7, BioLegend, no. 302306), CD23 (clone EBVCS-5, BioLegend, no. 338516), Human TruStain FcX (Fc Receptor Blocking Solution), BioLegend, no. 422302), and Brilliant Stain Buffer (BD Biosciences, no. 659611). FMO controls were prepared for each marker in the primary panel for CD3, CD19, CD20, CD23, and TIM-4-His. Single-stain controls were prepared for each antibody reagent to build the compensation matrix setup on the flow cytometer and sample analysis.

Flow analysis was performed by gating initially on SSC-H (height) versus FSC-H, followed by first singlet gating using SSC-A (area) and SSC-H parameters and second singlet gating using FCS-A and FSC-H. Sequentially, cells were gated based on live/dead staining the CD45^+^ population, followed by CD3^–^, then CD19^+^. Each of the gates were set based the respective FMO gating. Data were exported as FCS files and analyzed using FlowJo analysis software (BD Biosciences). Graphical reports were generated using FlowJo and Prism 9.3.1 (GraphPad Software, San Diego, CA).

### REC-1 and HCC827 tumor engraftment in NSG mice and CER-1236 T cell administration

All experimental procedures and protocols were approved by the Institutional Animal Care and Use Committee of Charles River Labs (Wilmington, MA). Animals were kept in a pathogen-free environment with a filtered air supply. Five- to 7-week-old female NSG mice (IL-2 receptor γ chain null NOD.Cg-Prkdcscid Il2rgtm1Wjl/SzJ) were obtained from Jackson Laboratory (Bar Harbor, ME).

For a disseminated *in vivo* model of MCL, NSG mice were engrafted i.v. via the lateral tail vein with 5e-6 REC-1 fLuc^+^ cells in 100 μL PBS without magnesium or chloride ions on day −2. After 24 h, on day −1, mice were randomized, then treated with 8 mg/kg ibrutinib or vehicle in drinking water. Mice were estimated to drink 4 mL per day, and ibrutinib formulated in 20% β-hydroxy cyclodextrin was diluted into drinking water in an amber bottle to provide a dose of 8 mg/kg based on mouse body weight. Vehicle-treated animals received an equivalent amount of 20% β-hydroxy cyclodextrin in their drinking water. Mouse weight and water intake were monitored twice per week, and ibrutinib was reformulated weekly based on changes in body weight and water intake. Animals were assessed daily for signs of GvHD, including hair loss, skin rash, skin thickening, and hunched posture. Forty-eight hours after REC-1 engraftment, on day 0, animals were infused i.v. via the lateral tail vein with 7.5e-6 CER-1236 T cells in 100 μL PBS without magnesium or chloride ions. Control mice were infused i.v. with untransduced T cells equivalent to the dose given to the 7.5e-6 CER-1236 T cell groups. CER-1236 T cells were manufactured from healthy donor leukopaks on a CliniMACS Prodigy instrument (Miltenyi Biotec, Bergisch Gladback, Germany).

Tumor burden was monitored twice per week using BLI measured on an Ami HTX imager (Spectral Instruments, Tucson, AZ) following an intraperitoneal injection of D-luciferin substrate solution (100 μL, 30 mg/mL). Peripheral blood was collected weekly by submandibular bleed into a K2-EDTA tube (BD, Franklin Lakes, NJ) and stored at −80°C. Mice were removed from the study when BLI reached 1 × 10^8^. At 16 days post T cell infusion, two to three animals were euthanized from CER-1236 or untransduced treated animals and liver, lungs, kidney, heart, and brain were collected and fixed in 10% neutral-buffered formalin for 24 h, then transferred to 70% ethanol. Organs were embedded in paraffin, sectioned, and IHC for anti-human CD3 or H&E staining was performed (Cureline Biopathology, Brisbane, CA). Images were taken at 40× or 4× using a BZ-X710 microscope (Keyence).

For evaluating CER-1236 T cell anti-tumor efficacy in a localized solid tumor model of NSCLC, NSG mice were subcutaneously injected (day −28) in the right flank with 3e-6 HCC827 target cells in a 100 μL volume of 1:1 mixture of Matrigel and PBS without magnesium or chloride ions. Once the tumor was palpable (average tumor volume of ∼80 mm^3^) animals were randomized into groups (five to eight animals) based on tumor volume (day −7). Two days before T cell infusion, mice were treated intraperitoneally with 100 μL of 0.5 mg/kg osimertinib (LC Laboratories, Woburn, MA) or vehicle control. Drug or vehicle i.p. administration was continued for 14 days on a QOD schedule. Twenty-eight days after the start of HCC827 engraftment (average tumor volume of ∼100 mm^3^), animals were infused i.v. via the lateral tail vein with 2.5e-6 CER-1236 T cells or untransduced T cell controls in 100 μL PBS without magnesium or chloride ions. Tumor burden was monitored twice per week with calipers until tumor volumes reached the euthanization threshold of 2,000 mm^3^. Tumors were measured along the longest axis, and perpendicular to the longest axis. Tumor volume was calculated using the formula V = ½(length × width^2^). Peripheral blood was collected every 2 weeks by submandibular bleed into a K2-EDTA tube (BD, Franklin Lakes, NJ) and stored at −80°C. At 21 days post T cell infusion, one animal from the T cell infusion groups was euthanized from CER-1236 or untransduced treated animals and tumors were collected and fixed in 10% neutral-buffered formalin for 24 h, then transferred to 70% ethanol. Tissue were embedded in paraffin, sectioned, and IHC for anti-human CD3 was performed (Cureline Biopathology, Brisbane, CA). Images were taken at 4× or 40× using a BZ-X710 microscope (Keyence).

### Evaluation of CER-1236 expansion in peripheral blood

To isolate genomic DNA from peripheral blood samples, samples were thawed and DNA was isolated using the Maxwell RSC Blood DNA kit (Promega, Madison, WI) using the Maxwell RSC benchtop automated DNA/RNA extraction instrument (Promega).

Total CER-1236 T cells present in the blood was determined using ddPCR. Test probes were designed against the unique CER-1236 TIM-4 and CD28 transmembrane domain junction, and control probes were designed against the HAT gene. The HAT gene primers and probes detect conserved regions of the gene present in both murine and human cells. For CER-1236 determination, the following primer/probe sets were used: CER-F: GGCATCCCTATGTCAATGAAG; CER-R: ACGGTGACAAGCAGGGAGTA; FAM-linked CER-probe: CTCTCAGTTTTGGGTCCTGG; HAT-F: TACGCTCTTTGCGACCGTAG; HAT-R: GGCACGTGTTATACTGCCAA; HEX-linked HAT-probe: ACCCAGACAAAACCCGGCCACGTGT. All primers and probes were obtained from IDT (Coralville, IA).

The ddPCR reactions were prepared with 1× ddPCR Supermix for probes (no dUTP; Bio-Rad, Hercules, CA), 0.4 μM of each primer, 0.2 μM of each probe, 50 U EcoRI-HF (New England Biolabs, Ipswich, MA), and 10 ng genomic DNA. The ddPCR droplets were generated using the QX200 Automated Droplet generator (Bio-Rad). Droplets were analyzed on the QX200 Droplet reader (Bio-Rad) using the direct quantification program. The following equation was used to determine copy number per μL of blood: (copies of CER-1236 per μL of reaction × total μL of reaction)/μL of genomic DNA used.

### Statistical analysis

All experimental data are presented as mean ± standard error of the mean or standard deviation, as indicated in figure legends. Statistical significance between two groups was determined using two-tailed Student’s t test. For multiple group comparison, One- or two-way ANOVA was used. A p value of less than 0.05 was considered statistically significant. Data were analyzed and presented with GraphPad Prism.

## Data Availability

The data that support the findings of this study are openly available at GEO (accession no. GSE232108). The authors confirm that the data supporting the findings of this study are available within the article and its supplemental information.

## Supplemental information

Document S1. Figures S1–S11

Table S1. Differentially expressed genes between CER-1236 and TIM-4 mutant CER-1251 T cells

Table S2. IPA pathway enrichment between CER-1236 and TIM-4 mutant CER-1251 T cells

Document S2. Article plus supplemental information
