# Supplementary material for: Chimeric TIM-4 receptor-modified T cells targeting phosphatidylserine mediates both cytotoxic anti-tumor responses and phagocytic uptake of tumor-associated antigen for T cell cross-presentation
Source: Mol Ther. 2023 May 16;31(7):2132–53. doi: 10.1016/j.ymthe.2023.05.009 (PMC10362418; doi:10.1016/j.ymthe.2023.05.009)
Supplement: Document S1. Figures S1–S11 [file mmc1.pdf]

YMTHE, Volume *31*

Supplemental Information

Chimeric TIM-4 receptor-modified T cells targeting phosphatidylserine mediates both cytotoxic anti-tumor responses and phagocytic uptake of tumor-associated antigen for T cell cross-presentation

Brandon Cieniewicz, Ankit Bhatta, Damoun Torabi, Priya Baichoo, Mike Saxton, Alexander Arballo, Linh Nguyen, Sunil Thomas, Harini Kethar, Phanidhar Kukutla, Omolola Shoaga, Bi Yu, Zhuo Yang, Maria Fate, Edson Oliveira, Hongxiu Ning, Lawrence Corey, and Daniel Corey

Supplemental Materials and Methods

Cell Production and Analysis

At indicated days during cell production, cell viability was measured using an NC-200 cell counter (Chemometec; Lillerod, Denmark). Cell counts were assessed at day 5 and 7 of cell production using an NC-200 cell counter to estimate total cells. Total cell number/initial cell number was used to determine fold expansion.

At the end of cell production, cells were washed and stained with Fixable Live/Dead dye (Invitrogen; Waltham, MA), anti-CD3 (Clone SK7; Biolegend; San Diego, CA), anti-CD4 (Clone RPA-T4; BD Biosciences; San Jose, CA), anti-CD8 (Clone RPA-T8; BD Biosciences), and PD-1 (Clone REA1165; Miltenyi Biotec). Cells were analyzed using a Cytoflex (Beckton Coulter; Brea, CA).

Plate-bound Phosphatidylserine-induced Cytokine Secretion

CER-1236 T cells or control cells were cultured on a plate coated with 5 μg/mL phosphatidylserine (Avanti Lipids; Alabaster, AL) in the presence or absence of 0.5 or 5 μM of C29, a TLR inhibitor (Selleckchem; Houston, TX) for 48h. Supernatant was collected from each well and cytokine secretion was assessed using the ELLA automated immunoassay system. Average for 2 technical duplicates ±SD is shown.

Bead-Based Phagocytosis Assay

PS-coated agarose beads of 0.5 diameter (Echelon Biosciences; Salt Lake City, UT) were washed with PBS containing 0.5% Tween 20 and centrifuged at 2500 xg and room temperature for 5 minutes. This was followed by 2 washes with PBS containing 100 U/mL penicillin/streptomycin. The beads were resuspended with 1 mL of media, sonicated in a water bath at room temperature for 5 minutes, and diluted with OpTmizer media. Diluted beads were added to untreated 96-well flat bottom plates at 5000 beads to yield respective cell-to-bead ratio of 5:1. Thawed or fresh CER-1236, CER-1251, or untransduced T cells were counted and diluted with OpTmizer media, and 25,000 live cells were added to the bead-containing plates. Samples were tested in triplicate per condition. Plates were incubated in a humidified 37°C chamber containing 5.0% CO_2 for 16h. To measure engulfment following cell incubation, samples of CER T cells and beads were stained with 1:100 anti-human CD3-fluorescein isothiocyanate (FITC; BioLegend; San Diego, CA) and 1:1000 fixable viability dye eFluor 780 (Thermo Fisher Scientific) on ice for 20 minutes. After staining, cells were washed

with pH 8 phosphate wash buffer (Fisher Scientific; Pittsburgh, PA). The pH 8 phosphate buffer was used to test the effect of quenching the pHrodo signal of extracellular beads that could be tethered to cell surfaces (6). Samples were acquired on the Cytoflex flow cytometer (Beckman Coulter). Parallel samples were imaged at 40x magnification using a Keyence BZ-X710 microscope (Osaka, Japan).

REC-1 Tumor Engraftment in NSG or NSG MHC dKO Mice and CER-1236 T-cell Administration

All experimental procedures and protocols were approved by the Institutional Animal Care and Use Committee of Charles River Labs (Wilmington, MA). Animals were kept in a pathogen-free environment with a filtered air supply. Five- to 7-week-old female NSG-(K^bD^b)^{null} (IA)^{null} mice (NOD.Cg-Prkdc^{scid} H2-K1^{tm1Bpe} H2-Ab1^{em1Mvw} H2-D1^{tm1Bpe} Il2rg^{tm1Wjl}/SzJ) or NSG (NOD.Cg-Prkdc^{scid} Il2rg^{tm1Wjl}/SzJ) were obtained from Jackson Laboratory (Bar Harbor, ME).

For a disseminated in vivo model of MCL, NSG MHC dKO or NSG mice were engrafted and monitored as described in main methods.

In NSG mice, peripheral blood was collected via cardiac puncture at 7 and 14 days after T cell infusion into K2-EDTA or citrate tubes. K2-EDTA tubes were submitted for CBC analysis (IDEXX Laboratories; Westbrook, ME) and citrate tubes were submitted for coagulation analysis (IDEXX Laboratories).

Evaluation of CER-1236 expansion in peripheral blood

Analysis was performed as described in main methods. The following equation was used to determine copy number per µg of DNA: CER probe counts*(1000000)/(Probe count for housekeeping gene*3.3pg/copy), where 3.3pg is the approximate weight of the human genome.

Measurement of PS exposure by flow cytometry

For evaluation of PS exposure on EGFR-mutation positive NSCLC H1975 and HCC827 cells, target cells were treated with the described doses of osimertinib. After 96 or 120 hours of drug treatment, target cells were harvested by enzymatic disassociation and proceeded to stain for PS exposure by flow cytometry as described in main methods.

Evaluation of CER-1236 effector function on EGFR-mutation positive NSCLC H1975 cells

H1975 NSCLC cells were pretreated for 24 hours with 100nM osimertinib, and EGFR inhibitor, or DMSO and then maintained in co-culture with 4.8nM osimertinib or DMSO in the presence of untransduced T cells of CER-1236 T cells at an effector: target (E:T) of 1:4. CER T-cell cytotoxic function was evaluated using the Incucyte live-cell analysis system. After co-culture, cytokine secretion was measured by automated ELISA. Proliferation was measured by flow cytometry using precision count beads to determine the absolute counts of viable T cells at the end of the co-culture.

Table S1. Differentially expressed genes between CER-1236 and TIM-4 mutant CER-1251 T cells.

Table S2. IPA pathway enrichment between CER-1236 and TIM-4 mutant CER-1251 T cells.

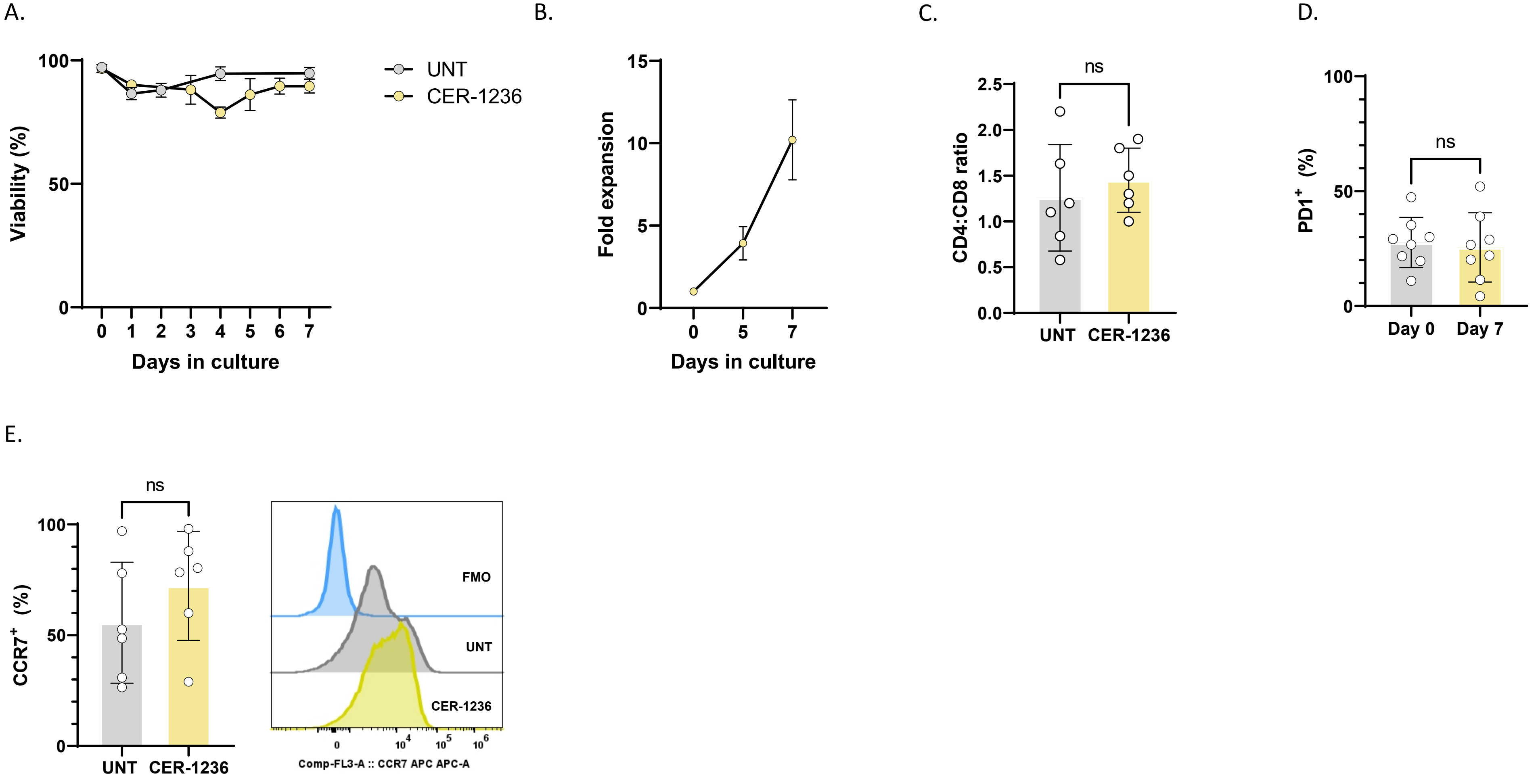

Figure S1. T cell characterization across different manufacturing runs. Untransduced T cells or CER-1236 T cells generated on the Prodigy system. (A) Cell viability was assessed throughout production using an automated cell counter. Average of 5 cell productions is shown ±SD. (B) CER-1236 T cells show robust expansion. At 5 and 7 days post production, cell counts were measured and total fold expansion was determined. The average of 5 cell productions ±SD is shown. (C) CD4 to CD8 ratio of CER-1236 cells is comparable to untransduced cells. CD4 to CD8 staining was measured in CD3+ cells by flow cytometry. Average of 6 cell productions ±SD is shown. (D) PD-1 levels are not altered during cell production. Levels of PD-1 at the start of cell production (day 0) and the end of cell production (day 7) were measured by flow cytometry. Average PD-1 positivity on CD3+ cells is shown ±SD. Statistics were determed by paired t-test. Ns = not significant. (E) Memory profile characterization showing high CCR7 positivity. Average of 6 cell productions ±SD are shown. Statistics were determed by paired t-test. Ns = not significant. Representative plots of CCR7 are shown on the right. Abbreviations: CER = chimeric engulfment receptor; UNT = untransduced.

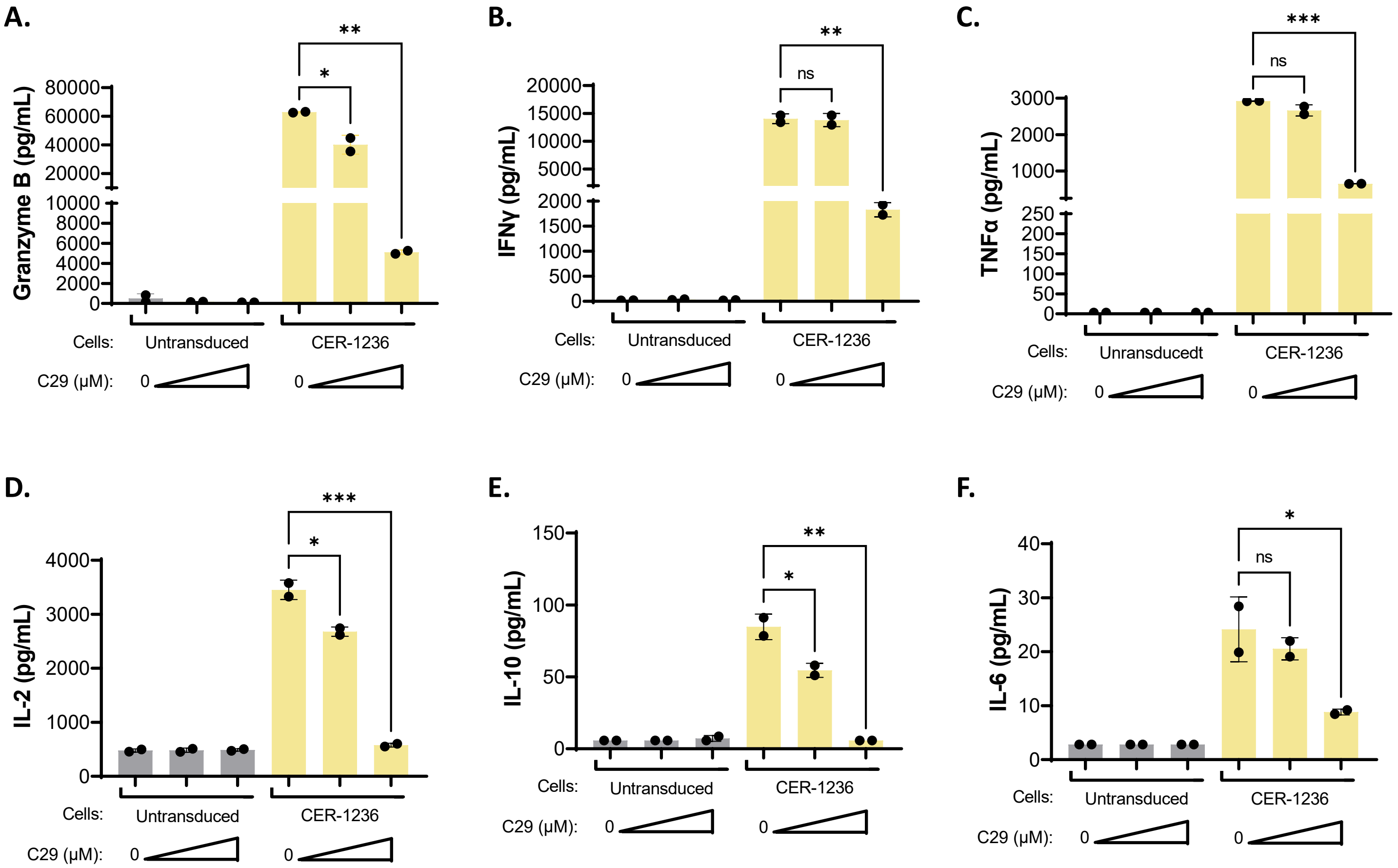

Figure S2. Effect of TLR2 inhibition towards cytokine secretion. Assessment of (A) granzyme B, (B) IFNγ, (C) TNFα, (D) IL-2, (E) IL-10 and (F) IL-6 secretion levels at 48h post plating in response to immobilized PS in the presence or absence of a small-molecule TLR2 inhibitor (C29) in two different doses: 5 μM and 0.5 μM. Untransduced cells were used as controls. N = 2 per treatment. Statistics were determined by one-way ANOVA with Sidak's multiple comparisons test. * = p>0.05, ** = p>0.01, *** = p>0.001, **** = p>0.0001. Abbreviations: CER = chimeric engulfment receptor; IFN = interferon; TNF = tumor necrosis factor; IL = interleukin.

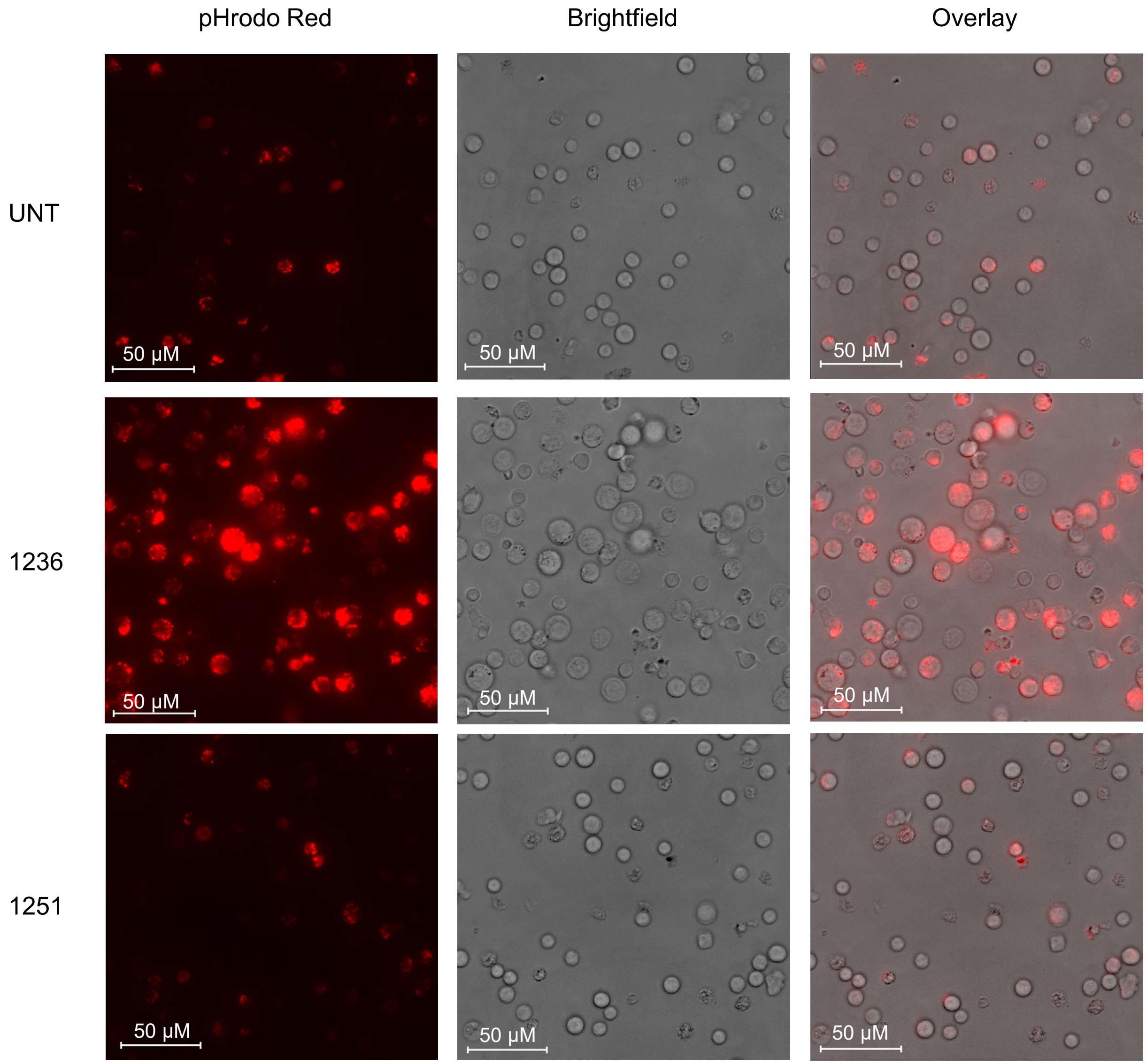

Figure S3. CER-1236 demonstrates target-dependent phagocytic uptake of PS-coated agarose beads. Fluorescence microscopic images (40x magnification) of indicated T cells cultured with pHrodo red beads at 40 h. (Top and bottom rows) Untransduced T cells and CER-1251, which harbor a mutant TIM-4 receptor, demonstrate minimal bead phagocytosis. (Middle row) CER-1236 T cells phagocytose agarose beads at high frequency and magnitude.

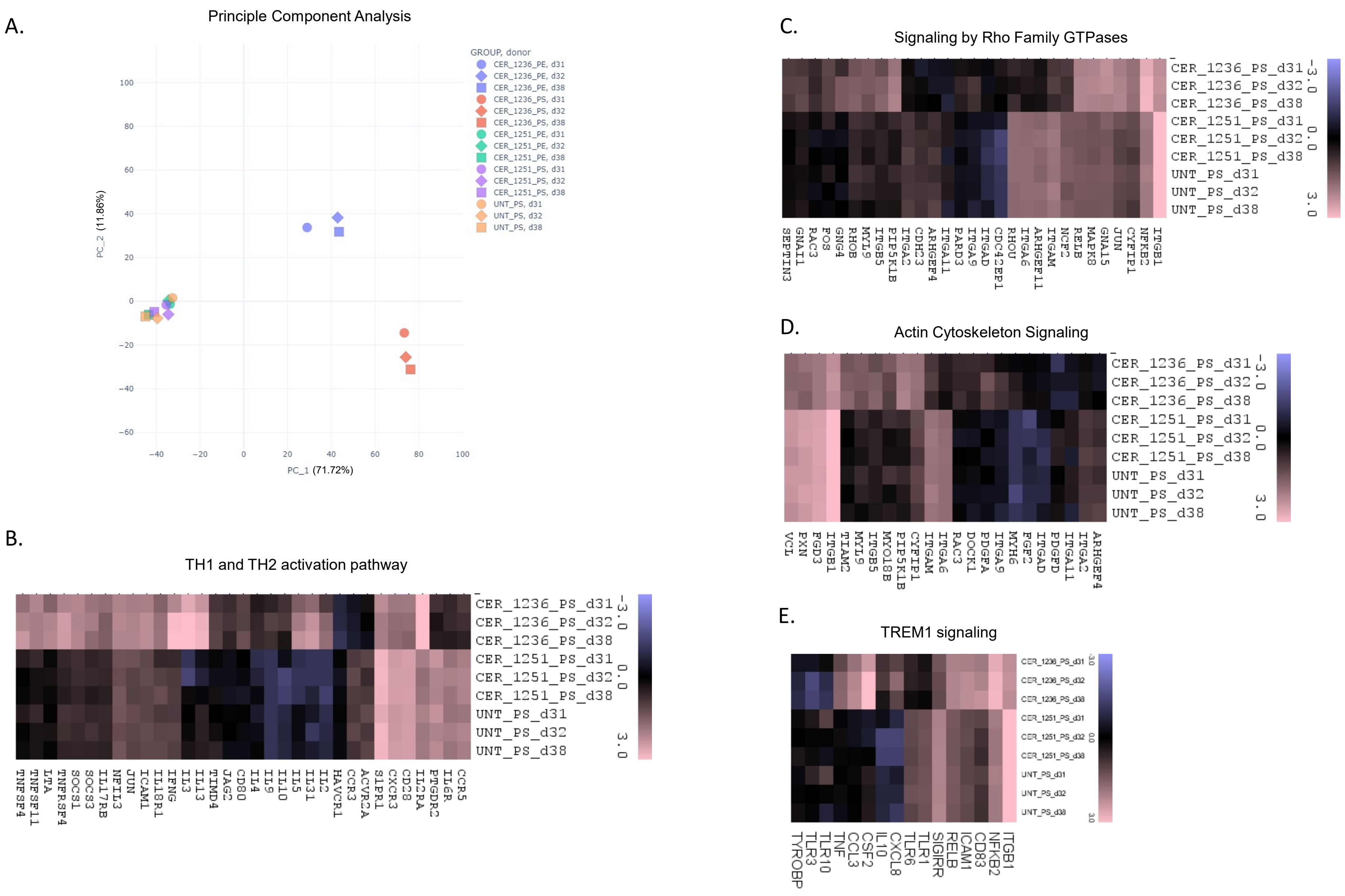

Figure S4. Transcriptional signatures of activated CER-1236 T cells indicate pathways enriched for key regulators of phagocytosis and cytotoxicity. (A) PCA plots of RNA-seq for CER-1236, CER-1251, or Untransduced T cells from three independent human donors. Cells were stimulated with immobilized PS or a related phospholipid PE for 24 h and then harvested for analysis. (B-E) Heat maps from IPA gene sets (FDR < 0.05, absolute fold change >4) from three donors 24 h post-stimulation, showing both upregulated and downregulated genes, expressed as log10(counts).

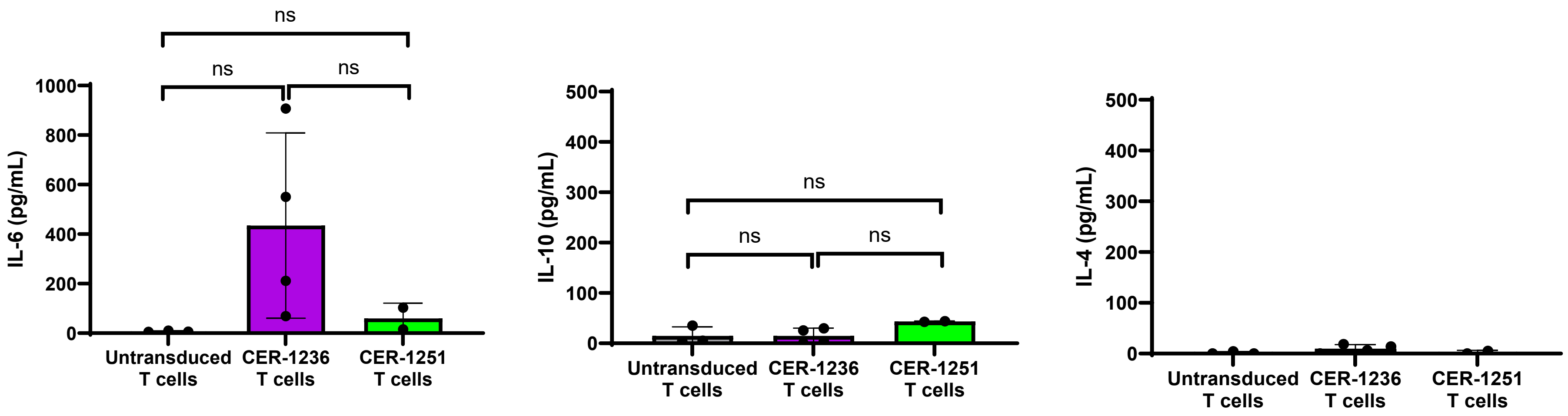

Figure S5. CER-1236 cytokine secretion after coculture with JeKo-1 TMEM30A KO MCL cells. CER-1236 were co-cultured with JeKo-1 mCherry+ TMEM30A KO cells at a 1:1 ratio for 120h. Untransduced cells were included as controls. After co-culture, cytokine secretion of of (A) IL-6, (B) IL-10, (C) IL-4 was measured by automated ELISA. Average cytokine secretion ±SD is shown (n=3 donors for untransduced, 4 donors for CER-1236, 2 donors for CER-1251). Statistics were analyzed using a 1-way ANOVA with Tukey's multiple comparisons test. ns = not significant. Abbreviations: CER = chimeric engulfment receptor; IL = interleukin.

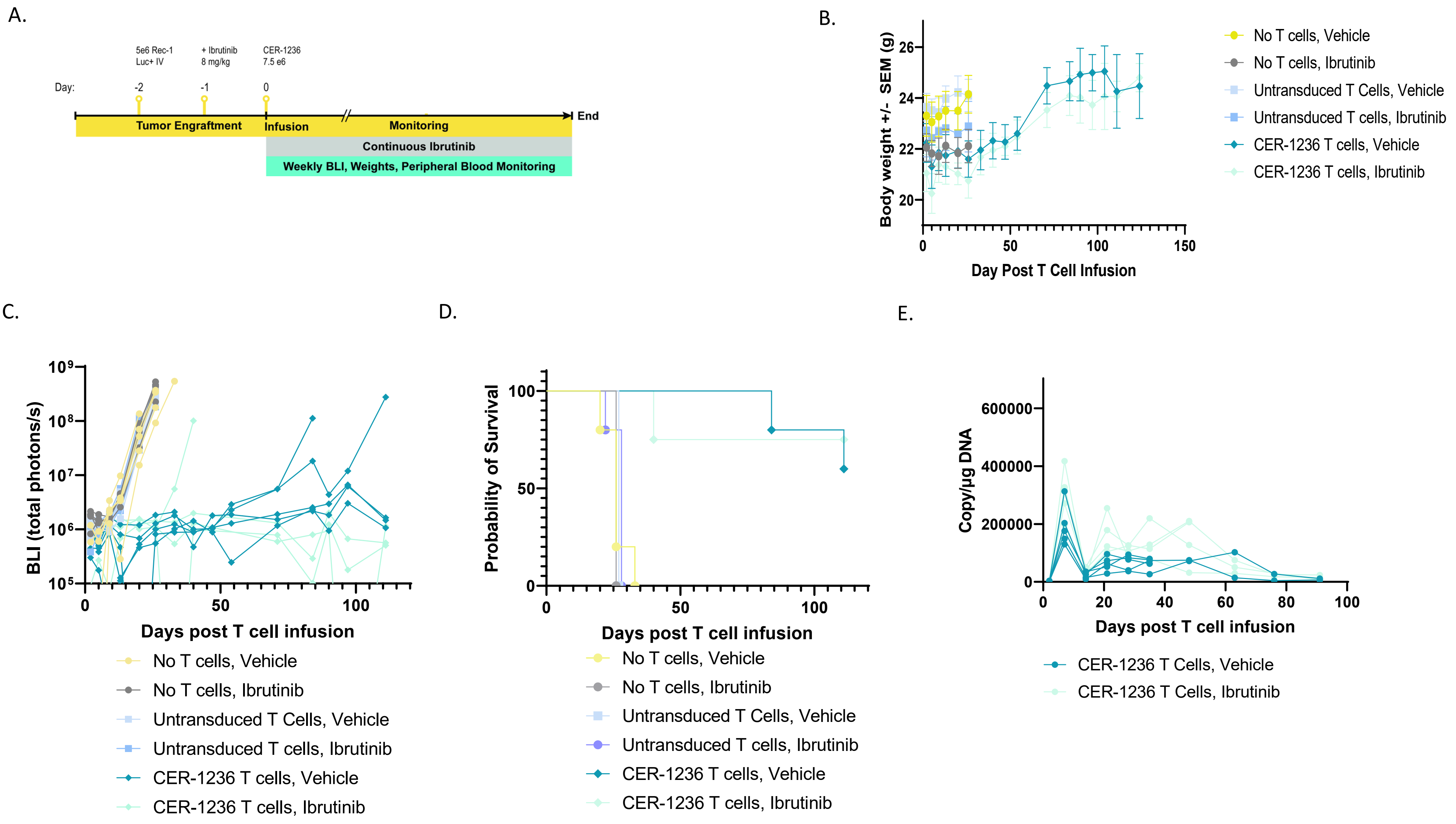

Figure S6. CER-1236 eliminates REC-1 xenografts in NSG MHC dKO mice. (A) Schematic of experiment design. (B) CER-1236 treatment does not affect body weight. Average body weight of mice in each group ±SEM is shown. (C) CER-1236 treatment eliminates REC-1 tumors. Tumor growth was monitored by BLI imaging of mice on the indicated days post T cell transfusion. Individual BLI curves for each mouse are shown. (D) CER-1236 treatment improves survival in NSG mice with REC-1 MCL xenografts. Probability of survival shown for mice treated with CER-1236 or control groups. Mice were removed from study upon reaching BLI maximum of 1e8 photons/s. (E) CER-1236 expansion peaks at 7 days post administration and decreases after. DNA was isolated from peripheral blood and frequency of CER-1236 T cells was determined by ddPCR using a CER-specific primer/probe set. Copy/μg of DNA was determined by CER probe counts*(1000000)/(Probe count for housekeeping gene*3.3). Longitudinal analysis is shown for each mouse in the indicated groups.

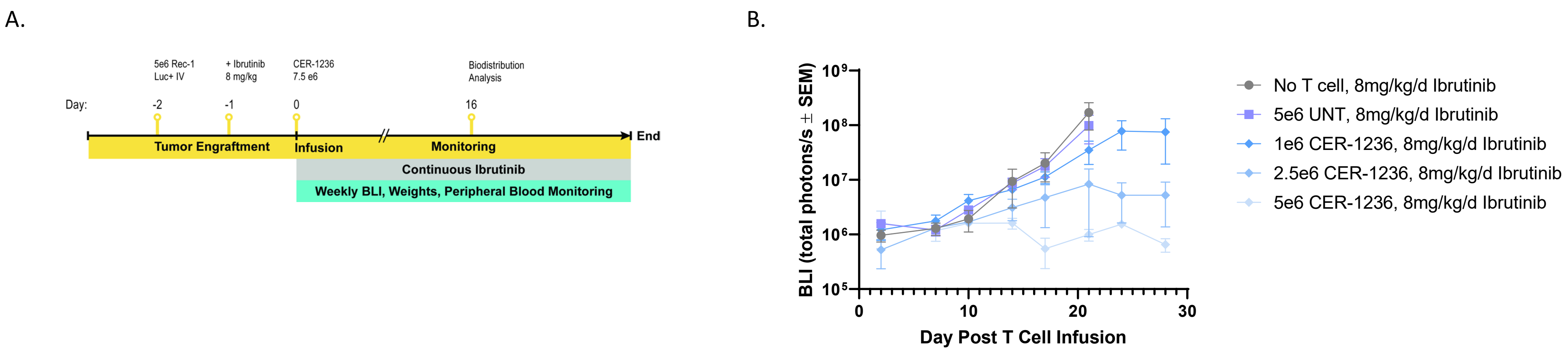

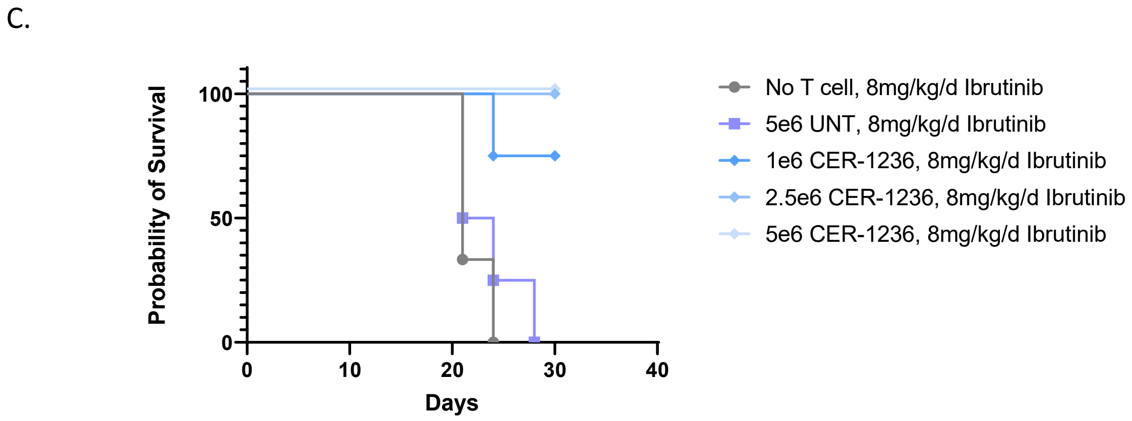

Figure S7. Evaluation of effective CER-1236 cell dose in NSG MHC dKO mice. (A) Schematic of experiment design. CER-1236 T cells were infused into REC-1-bearing NSG MHC dKO animals at ascending doses to evaluate anti-tumor responses. (B) BLI imaging of mice on the indicated days after receiving 1e6, 2.5e6, and 5e6 CER-1236 T cells. (C) Survival curves for mice treated with increasing doses of CER-1236 T cells.

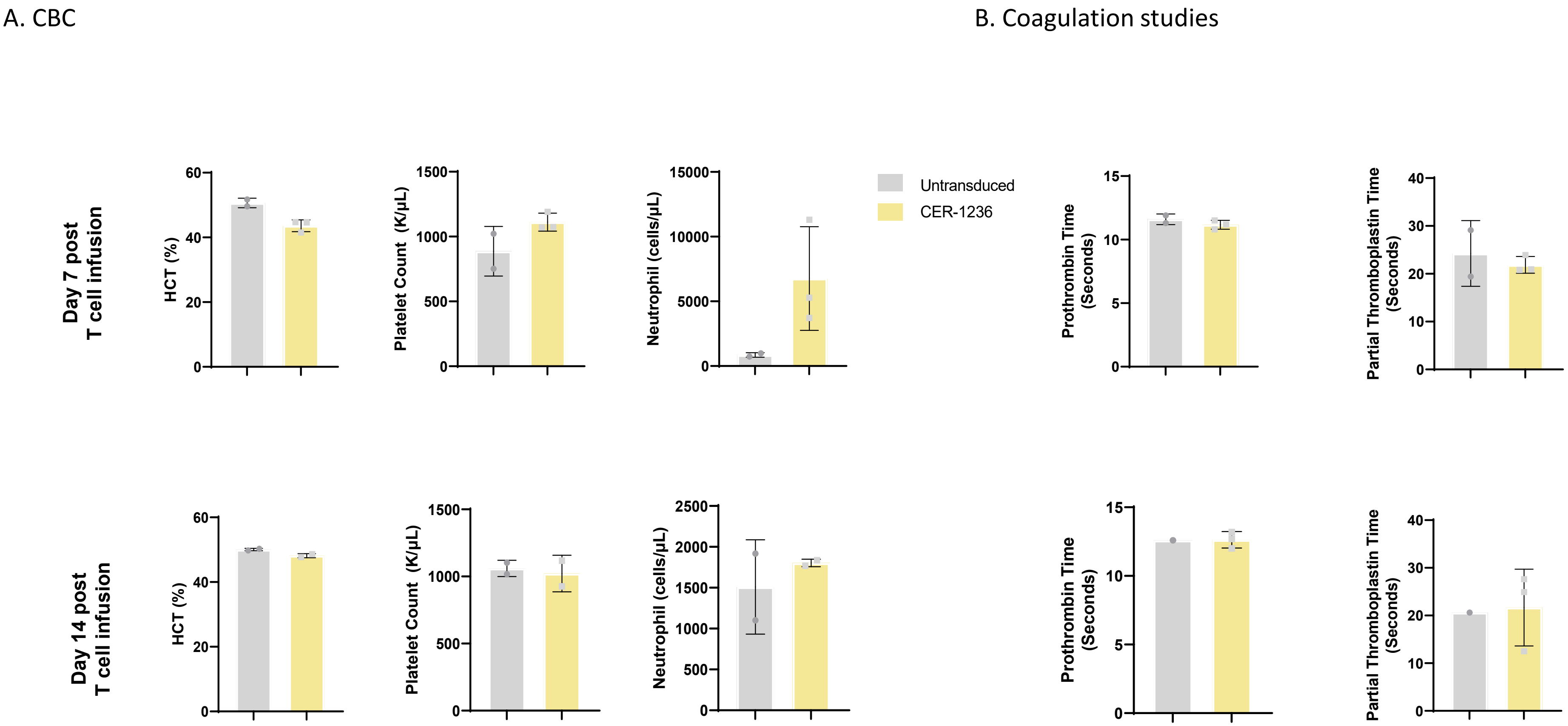

Figure S8. Hematologic indices in NSG studies. (A) CBC profiling at days 7 and 14 in untransduced and CER-1236 T cell groups with concurrent BTK inhibitor therapy (ibrutinib). No difference in hemoglobin, platelet, and neutrophil counts between groups. (B) PT-INR and PTT coagulation studies at day 7 and 14 in untransduced and CER-1236 T cell groups with concurrent BTK inhibitor therapy (ibrutinib). No prolongation in bleeding times were observed in either group. Average ±SD is shown.

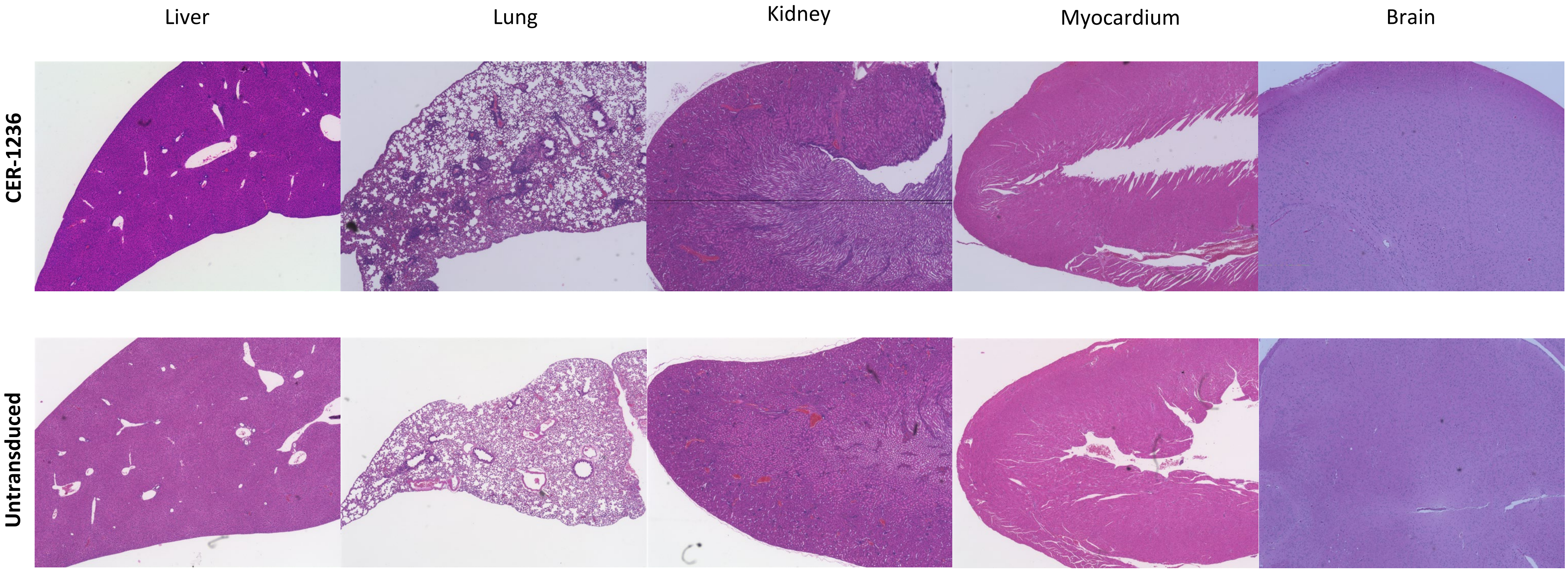

Figure S9. H & E staining of murine tissues shows no gross morphological defects. (A) H&E staining of select organs. At d16 post T cell transfusion, untransduced CER-1236 treated animals receiving Ibrutinib were sacrificed and organs were removed for H & E staining. Representative images of 4x images of liver, lung, kidney, myocardium, and brain are shown.

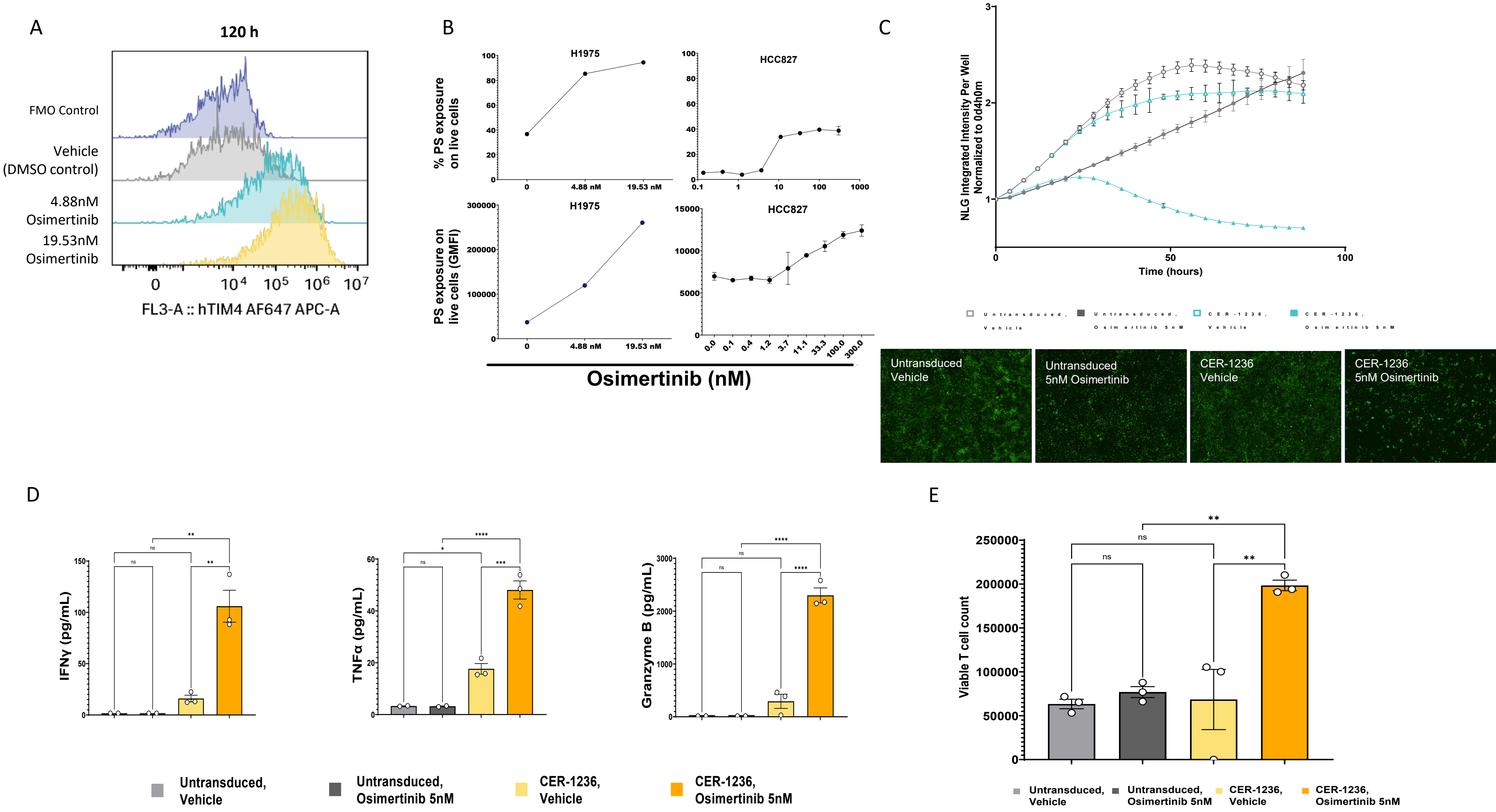

Figure S10. CER-1236 mediates cytotoxicity, cytokine secretion, and proliferation against EGFR-mutation positive NSCLC H1975 cells, augmented by osimertinib, an EGFR inhibitor. (A) Histogram overlays of PS exposure on viable H1975 NSCLC targets after 120h of osimertinib treatment. (B) PS detection on viable H1975 and HCC827 targets by flow cytometry after osimertinib treatment for 120h and 96hr respectively. (C) CER-1236 co-cultures demonstrates enhanced tumor elimination in combination with osimertinib treatment. CER-1236 were co-cultured with H1975 targets at a E:T ratio of 1:4 for 120 h. untransduced T cells were included as controls. (bottom) Representative IncuCyte image overlays of phase/contrast and green channel taken at 120h post co-culture demonstrating H1975 target abundance or lack thereof from co-cultures. (D) CER-1236 T cells secrete effector cytokines in response to H1975 target cells. Co-culture with osimertinib treated H1975 targets led to further enhancement of secretion of IFNγ, TNFα and Granzyme B compared to untreated targets. Average cytokine secretion ±SEM is shown from 3 technical triplicates (n=1). Statistics were determined using a 1-way ANOVA with Tukey's multiple comparisons test. * = p<0.05, ** = p<0.01, *** = p<0.001, **** = p<0.0001. (E) Enhanced proliferation of CER-1236 T cells co-cultured with osimertinib treated targets, compared to untreated target co-cultures of CER-1236 and untransduced T cell co-cultures. Average viable T cell counts ±SEM is shown from technical triplicates (n=1). Statistics were determined using a 1-way ANOVA with Tukey's multiple comparisons test. ** = p<0.01. Abbreviations: PS = phosphatidylserine; NSCLC = non-small cell lung cancer.

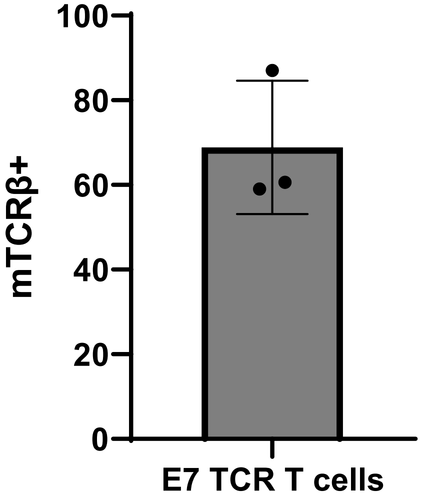

Figure S11. E7 TCR T cells have high transduction. Expression of the E7 TCR in cross presentation assays was assessed by flow cytometry. Transduction efficiency was determined by murine TCRβ detection in CD3+ cells. Data represents 3 independent transductions, ± SD. Abbreviations: TCR = T cell receptor.
